# 29th Annual GP2A Medicinal Chemistry Conference

**DOI:** 10.3390/ph14121278

**Published:** 2021-12-07

**Authors:** Jean-Jacques Helesbeux, Laura Carro, Florence O. McCarthy, Vânia M. Moreira, Francesca Giuntini, Niamh O’Boyle, Susan E. Matthews, Gülşah Bayraktar, Samuel Bertrand, Christophe Rochais, Pascal Marchand

**Affiliations:** 1 Univ. Angers, SONAS, SFR QUASAV, F-49000 Angers, France; jean-jacques.helesbeux@univ-angers.fr; 2 School of Pharmacy, University College London, London WC1N 1AX, UK; l.carro@ucl.ac.uk; 3 School of Chemistry, Analytical and Biological Chemistry Research Facility, University College Cork, College Road, T12 K8AF Cork, Ireland; f.mccarthy@ucc.ie; 4 Laboratory of Pharmaceutical Chemistry, Faculty of Pharmacy, University of Coimbra, Pólo das Ciências da Saúde, Azinhaga de Santa Comba, 3000-548 Coimbra, Portugal; vmoreira@ff.uc.pt; 5 Center for Neuroscience and Cell Biology, Faculty of Medicine, University of Coimbra, Rua Larga, 3004-504 Coimbra, Portugal; 6 School of Pharmacy and Biomolecular Sciences, Byrom Street Campus, Liverpool John Moores University, Liverpool L3 3AF, UK; F.Giuntini@ljmu.ac.uk; 7 School of Pharmacy and Pharmaceutical Sciences, Panoz Institute, Trinity College Dublin, D02 R590 Dublin, Ireland; nioboyle@tcd.ie; 8 School of Pharmacy, University of East Anglia, Norwich Research Park, Norwich NR4 7TJ, UK; susan.matthews@uea.ac.uk; 9 Department of Pharmaceutical Chemistry, Faculty of Pharmacy, Ege University, Izmir 35100, Turkey; gulsah.bayraktar@ege.edu.tr; 10 Institut des Substances et Organismes de la Mer, ISOmer, Nantes Université, UR 2160, F-44000 Nantes, France; samuel.bertrand@univ-nantes.fr; 11 UNICAEN, CERMN (Centre d’Etudes et de Recherche sur le Médicament de Normandie), Normandie Univ., F-14032 Caen, France; christophe.rochais@unicaen.fr; 12 Cibles et Médicaments des Infections et du Cancer, IICiMed, Nantes Université, UR 1155, F-44000 Nantes, France

**Keywords:** pharmaceutical chemistry, medicinal chemistry, drug design, chemical tools, chemical biology, molecular pharmacology

## Abstract

The 29th Annual GP_2_A (Group for the Promotion of Pharmaceutical chemistry in Academia) Conference was a virtual event this year due to the COVID-19 pandemic and spanned three days from Wednesday 25 to Friday 27 August 2021. The meeting brought together an international delegation of researchers with interests in medicinal chemistry and interfacing disciplines. Abstracts of keynote lectures given by the 10 invited speakers, along with those of the 8 young researcher talks and the 50 flash presentation posters, are included in this report. Like previous editions, the conference was a real success, with high-level scientific discussions on cutting-edge advances in the fields of pharmaceutical chemistry.

## 1. Aim and Scope of the Meeting

The GP_2_A (Group for the Promotion of Pharmaceutical chemistry in Academia) network consists of researchers in the field of medicinal chemistry working in universities and research institutes across Europe. It was established in 1992 with the aim of bringing together researchers to exchange ideas and expertise and to facilitate a friendly collaborative networking environment. Historically, it has included members from France, Spain, Portugal, Ireland, and the United Kingdom (the “Atlantic Arc”). More recently, the network has expanded both geographically and in terms of research fields, and it now includes researchers with scientific expertise ranging from physical and pharmaceutical chemistry to molecular pharmacology.

The annual GP_2_A conference host city alternates between France and another country and brings together senior scientists and those starting in their scientific careers. Moreover, the network facilitates short visits between member laboratories across the network. A consistent goal of the annual conference is to provide young researchers, postdoctoral researchers, and PhD students an opportunity to present their work to an international audience through either poster presentation or by sharing the stage with invited speakers.

The GP_2_A network aims to embrace the multidisciplinary nature of drug discovery and chemical biology, including topics that cover a broad range of interest in both pharmaceutical chemistry and interfacing disciplines. These comprise infectious and neurodegenerative diseases, chemoprevention of cancer, approaches to target and hit identification, and optimization of drug candidates, among others. Other topics include natural products, inflammatory diseases/pain, and the application of structural chemoinformatics and new synthetic concepts.

Cutting-edge advances in the diverse fields of applied chemistry towards novel health solutions were disseminated by 10 internationally recognized experts from inside and beyond the network. In addition, 8 young researcher communications and 50 flash poster presentations were considered for oral and poster presentation prizes, respectively. The 68 individual topics and presentations covered in the congress afforded opportunities for outstanding discussions and future collaborations.

## 2. Keynote Lectures

### 2.1. From Activity-Based Protein Profiling to the Optimization of Covalent Inhibitors Targeting Dipeptidyl Peptidases (Kl01)

**Rui Moreira**

Research Institute for Medicines (iMed.ULisboa), Faculty of Pharmacy, Universidade de Lisboa, Av. Prof. Gama Pinto, 1649-003 Lisbon, Portugal; rmoreira@ff.ulisboa.pt

**Figure 1:**
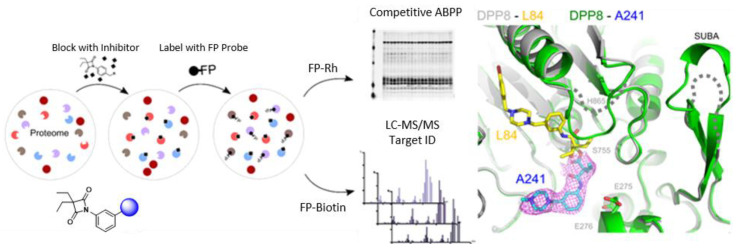
Identification of DDP8/9 inhibitors using activity-based protein profiling.

Dipeptidyl peptidases 8 (DPP8) and 9 (DPP9) are intracellular members of the structure homolog (DASH) sub-family of serine proteases that cleave polypeptide substrates after proline residues. Accessing chemical matter able to discriminate DPP8 or DDP9 engagement could significantly illuminate the biology of these proteases and allow further studies in a systems biology context. Indeed, evidence suggests their important role as checkpoints for inflammasomes and modulation of the immune system. Using comprehensive chemical proteomics profiling, we were able to deconvolute the hidden pharmacology of 4-oxo-β-lactams, showing that these covalent inhibitors potently and distinctively engage DPP8/9 (Figure 1). Importantly, by collecting high-resolution X-ray crystal data, we characterized to the atomic level a hitherto unknown mode of inhibition. Our findings enable, for the first time, the development of specific DPP8 or DPP9 effectors, which may become suitable for forthcoming medicinal chemistry and translational applications.

### 2.2. Programming Molecules for Therapeutic Applications (KL02)

**Sébastien Papot**

University of Poitiers, UMR 7285 (IC2MP), 4 rue Michel Brunet, 86022 Poitiers, France; sebastian.papot@univ-poitiers.fr

The rise of chemical biology has led to the development of sophisticated molecular devices designed to perform specific tasks within living systems. Most of these molecules have built into their structure a “chemical program” that determines their behavior during their interaction with biological environments. Thus, such molecular systems can be programmed to explore or manipulate the processes of life.

**Figure 2:**
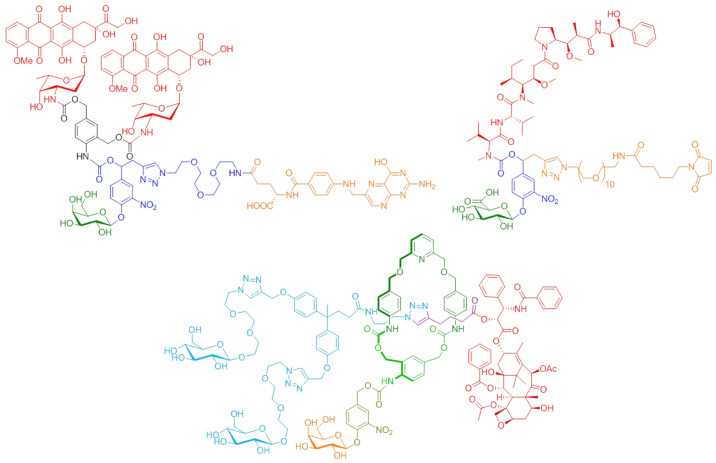
Self-immolative platforms.

Within this framework, we developed various molecular devices programmed for the selective delivery of anticancer drugs (Figure 2). These functional systems were designed to allow: (1) the transport in the body of potent anticancer agents in an innocuous manner toward safe tissues, (2) the efficient recognition of malignant specificities located either at the surface of cancer cells or in the tumor microenvironment, and (3) the controlled release of the parent drug exclusively at the tumor site. Such compounds include programming components such as self-immolative linkers, chemical amplifiers, self-opening macrocycles, enzyme-responsive biorthogonal triggers, and so on, which pilot the process of drug release in a stringently controlled fashion [1,2,3,4,5,6,7,8,9].

### 2.3. From Impurity Profiling to Purified Metabolites: Drug Discovery Supported by Analytical and Preparative Supercritical Fluid Chromatography (KL03)

**Caroline West**

University of Orléans, ICOA, CNRS UMR 7311, rue de Chartres, 45067 Orléans, France; caroline.west@univ-orleans.fr

**Figure 3:**
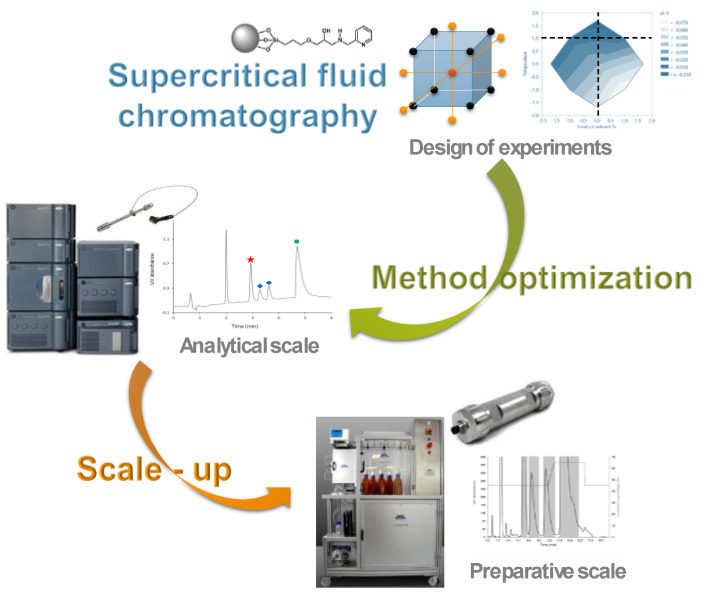
General applications of SFC.

Supercritical fluid chromatography (SFC) nowadays mostly relates to a chromatographic separation technique where the mobile phase is composed of pressurized carbon dioxide and a co-solvent. Chiral SFC has been largely employed for two decades in pharmaceutical industries, especially for its economic and ecological advantages at the preparative scale [10]. Achiral SFC, however, was not so frequently used, up until recent years. The introduction, by several manufacturers, of analytical SFC instruments that are better fitting to current expectations of chromatographers has significantly contributed to a renewed interest in this most versatile technique (Figure 3).

In this presentation, I will illustrate the interest in SFC in a drug discovery context with several examples. First, the interest in SFC as a complementary method to reversed-phase HPLC to achieve impurity profiling of drug candidates will be given [10,11]. Then, the interest in an easy transfer from analytical-scale to preparative-scale will be demonstrated with the example of drug metabolites produced via stressful degradation of an active pharmaceutical ingredient (API) [12]. Finally, while the potential of SFC for non-polar and moderately polar chemicals is usually well accepted, the applicability of carbon-dioxide-based mobile phases to more polar species such as glycosylated flavonoids will be demonstrated.

### 2.4. Drug-Loaded Polymeric Systems as a Promising Tool for Cancer Management (KL04)

**Catalin Zaharia ^1^, Ionut-Cristian Radu ^1^, Bianca Galateanu ^2^, Eugenia Tanasa ^1^, Madalina Necolau ^1^**

^1^ Advanced Polymer Materials Group, Politehnica University of Bucharest, 1-7 Gh. Polizu Street, 011061 Bucharest, Romania.

^2^ Department of Biochemistry and Molecular Biology, University of Bucharest, 91-95 Splaiul Independentei Street, 050095 Bucharest, Romania; zaharia.catalin@gmail.com

Technological advances led to the development of new innovative drug delivery systems. Nanotechnology provides a better safety profile against drugs with high toxic potential, and these nanoforms can be directed to act specifically at the target tissue by active as well as passive means [13]. Polymeric nanoparticulate systems from biodegradable and biocompatible polymers are interesting options for controlled drug delivery and drug targeting, especially in cancer therapy [14]. Cancer is a disease characterized by the uncontrolled growth and spread of abnormal cells and is still the second most common cause of death globally. Actual therapy for cancer includes surgery, radiation, hormone therapy, and chemotherapy. Nanoparticles are a valuable solution in oncology due to their ability of targeted delivery overcoming limitations of conventional chemotherapy, which include undesirable biodistribution, cancer cell drug resistance, and severe systemic side effects.

There are many types of nanoparticles which could be employed for cancer management. This includes dendrimers, liposomes, polymeric nanoparticles, micelles, protein NPs, lipid NPs, and so on. Among them, natural polymers based on proteins like silk fibroin and sericin are interesting systems with a huge potential in oncology. Here, we discuss the development of drug delivery systems based on silk fibroin and silk sericin loaded with various antineoplastic drugs. Protein nanoparticles were prepared by nanoprecipitation and then loaded with biological molecules. The chemistry, structure, morphology, and size distribution of nanocarriers were investigated by Fourier-transformed infrared spectroscopy (FTIR-ATR), scanning electron (SEM) and transmission electron microscopy (TEM), and dynamic light scattering (DLS). In vitro drug release assays were also performed in PBS solution at various pH values. The biological investigation via different cancer cell lines (breast and colorectal cancer) revealed a high activity of nanocarriers in cancer cells by inducing significant DNA damage.

**Acknowledgments:** This work was supported by a grant from the Ministry of Research, Innovation and Digitization, CNCS/CCCDI–UEFISCDI, project number PN-III-P4-ID-PCE-2020-1448, within PNCDI III.

### 2.5. Discovery of Small Molecules to Manipulate Cell Fate In Vivo: Towards New Therapies for Degenerative Diseases (KL05)

**Angela J. Russell**

Departments of Chemistry and Pharmacology, University of Oxford, Chemistry Research Laboratory, Mansfield Road, Oxford OX1 3TA, UK; angela.russell@chem.ox.ac.uk

With an increasingly ageing population, chronic diseases including cancers, dementia, and heart failure are placing a huge demand on society and healthcare services. Regenerative medicine approaches seek to transform healthcare management strategies, improving outcomes for patients suffering from degenerative diseases. Over the past two decades, the majority of studies have been focused on the transplantation of therapeutic cells. Several thousands of clinical trials have been conducted involving cell transplantation, and while there have been signs of efficacy in some cases, major hurdles exist to the routine adoption of such therapies in the clinic. Moreover, we now understand that in most cases, these cells act not as a cell replacement therapy, but rather through the stimulation of endogenous repair pathways already present within the body. This has opened a whole new avenue of research in the development of agents to directly stimulate these tissue repair and regeneration processes in the treatment of chronic degenerative diseases and injury, negating the need for cell transplantation [15].

The field of drug discovery for regenerative medicine will be introduced, and the impact this is beginning to have on the diseases of ageing described. Our own research in the discovery of small molecules to modulate utrophin for the treatment of the muscle degenerative disease Duchenne muscular dystrophy will be described, as well as translation of the first-generation utrophin modulator to the clinic and deconvolution of its molecular target and mechanism [16]. Our extension of this approach into regenerative medicine will then be described, exemplified by the discovery of small molecules to stimulate neurogenesis in vitro and in vivo [17].

### 2.6. Understanding Potent Small-Molecule Modulation of TRPC1/4/5 Channels (KL06)

**Robin S. Bon ^1^, Claudia C. Bauer ^1^, Aisling Minard ^1,2^, Isabelle B. Pickles ^1,2^, David J. Wright ^1^, David J. Beech ^1^, Stephen P. Muench ^3^, Katsuhiko Muraki ^4^, Stuart L. Warriner ^2^, Megan H. Wright ^2^**

^1^ Leeds Institute of Cardiovascular and Metabolic Medicine, Leeds LS2 9JT, UK

^2^ School of Chemistry, Leeds LS2 9JT, UK

^3^ School of Biomedical Sciences, Leeds LS2 9JT, UK

^4^ Dept. of Cellular Pharmacology, Aichi Gakuin University, 1-100 Kusumoto, Chikusa, Nagoya 464-8650, Japan; r.bon@leeds.ac.uk

TRPC proteins form tetrameric, non-selective cation channels permeable by Na^+^ and Ca^2+^ [18]. TRPC channels may consist of homomers or heteromers of subunits, each with their own characteristics and functions. Our research focuses on channels formed by the closely related TRPC4 and TRPC5 proteins—including heteromeric TRPC1/C4 and TRPC1/C5 channels. These channels are receiving increasing attention from both academia and industry as potential drug targets for the treatment of, for example, cancer, renal disease, cardiovascular remodeling and inflammation, complications of diabetes, and disorders of the central nervous system.

**Figure 4:**
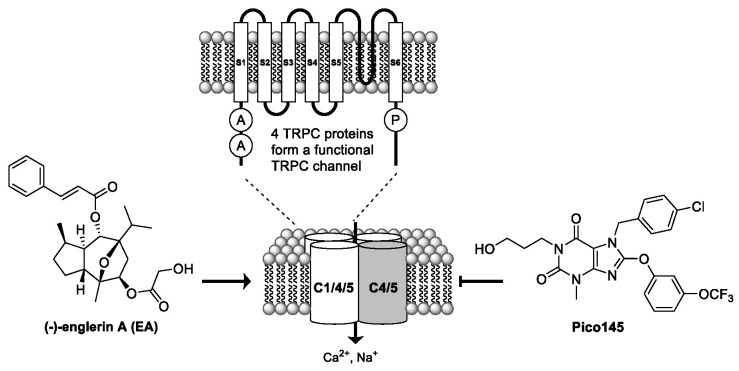
(-)-Englerin A (EA) and Pico145 are the most promising TRPC1/4/5 modulators.

Currently, the best TRPC1/4/5 activator is the natural product (-)-englerin A (EA) (Figure 4). The best inhibitors are xanthines, such as Pico145, which can be used in cells, tissues, and animals, and distinguishes between different TRPC1/4/5 tetramers [18]. The mechanisms by which EA and xanthines modulate TRPC1/4/5 channels—and can distinguish between closely related channels—is not known.

We hypothesize that EA and xanthines act via one or multiple distinct binding sites accessible from the extracellular side of the plasma membrane [19]. We will present the use of an integrated approach to understanding the mode-of-action of EA, xanthines, and endogenous modulators on TRPC1/4/5 channels. This includes the use of structure–activity relationship studies, the use of chimaeras and mutant channels in structure–function studies, the development and application of photoaffinity probes to test target engagement and identify binding sites, and the determination of high-resolution (2.8–3.1 Å) cryo-EM structures of TRPC5 channels in complex with different modulators.

### 2.7. Fragment-Based Approaches to Inhibit Mycobacterium Tuberculosis Targets (KL07)

**Marion Flipo ^1^, Baptiste Villemagne ^1^, Priscille Brodin ^2^, Benoit Deprez ^1^, Alain Baulard ^2^, Nicolas Willand ^1^**

^1^ Univ. Lille, Inserm, Institut Pasteur de Lille, U1177-Drugs and Molecules for living Systems, F-59000 Lille, France

^2^ Univ. Lille, CNRS, Inserm, CHU Lille, Institut Pasteur de Lille, U1019–UMR 9017-CIIL-Center for Infection and Immunity of Lille, F-59000 Lille, France; marion.flipo@univ-lille.fr

Tuberculosis, a disease caused by Mycobacterium tuberculosis (M. tb), remains the leading cause of death from a single infectious agent, killing 1.5 million people each year. The treatment of this disease involves a multidrug chemotherapy regimen often associated with serious side effects. Moreover, the growing threat of multidrug-resistant strains of M. tb stresses the need for alternative therapies.

In recent years, fragment-based drug design has emerged as an alternative approach to high-throughput screening to identify inhibitors of bacterial targets. This approach relies on the screening of compounds with low molecular weight (<300 Da) and high solubility, which makes them attractive for the aim of penetrating the thick and poorly permeable M. tb cell envelope.

Mycolic acids are very long chain fatty acids playing an essential role in the architecture and permeability of the bacterial wall. The biosynthesis of mycolic acids involves two fatty acid synthase (FAS) systems. Several antibiotics target the FAS-II cycle, which is composed of four enzymes (MabA, HadAB/BC, InhA, and KasA/B). InhA is inhibited by isoniazid and ethionamide, while no specific inhibitors of MabA have yet been reported. We used fragment-based approaches to target the FAS-II system, either by targeting MabA or by boosting ethionamide activity.

A fragment-based screening allowed the discovery of the first inhibitors of MabA (FabG1), an enzyme belonging to the FAS-II elongation system. The screening of our 1280 fragment-library on MabA, using a new LC–MS/MS based enzymatic assay, allowed the identification of several families of inhibitors. One of these chemical series was optimized and the binding of the compounds to MabA was confirmed by 19F ligand-observed NMR experiments [20].

A fragment-growing strategy was also conducted to discover ligands of EthR, a mycobacterial transcriptional regulator involved in the control of the bioactivation of the second-line drug ethionamide. These EthR inhibitors were able to boost ethionamide activity ten times at low nanomolar concentrations in vitro. The exploration of the structure–activity and structure–property relationships led to the identification of the first fragment-based ethionamide booster, which proved to be active in vivo in an acute model of tuberculosis infection [21].

### 2.8. Chemical Approaches for the Study and Inhibition of Bacterial L,D-Transpeptidases (KL08)

**Saidbakhrom Saidjalolov, Emmanuelle Braud, Mélanie Etheve-Quelquejeu**

UMR 8601, CNRS, Team: Chemistry of RNA, Nucleosides, Peptides & Heterocycles, University of Paris, 75006 Paris, France; melanie.etheve-quelquejeu@parisdescartes.fr

The L,D-transpeptidases are the main peptidoglycan cross-linking enzymes in mycobacteria and are promising targets for the development of new antituberculosis drugs, as shown by recent results obtained in vitro [22] and in a phase II clinical evaluation of the triple combination comprising a carbapenem, a β-lactamase inhibitor, and amoxicillin [23]. These enzymes catalyze the cleavage of the L-Lys3-D-Ala4 or DAP3-D-Ala4 bond of an acyl donor containing a tetrapeptide stem and the formation of the L-Lys3→L-Lys3 or DAP3→DAP3 cross-links. We developed strategies to better understand their role in peptidoglycan synthesis. In this conference, I will present the design and the synthesis of chemical tools to study L,D-transpeptidases in *Enterococcus faecium* and *Mycobacterium tuberculosis*. This project relies on the synthesis of peptide stems of peptidoglycan precursors and the development of a sensitive post-derivatization assay for their cross-linking by L,D-transpeptidases [24]. I will also present the synthesis of a series of β-lactam-peptides conjugates, and finally, the use of click and release chemistry for activity-based proteins purifications.

### 2.9. Metal Complexes in Medicinal Chemistry (KL09)

**Gilles Gasser**

Chimie ParisTech, PSL University, CNRS, Institute of Chemistry for Life and Health Sciences, Laboratory for Inorganic Chemical Biology, 75005 Paris, France; gilles.gasser@chimieparistech.psl.eu

**Figure 5:**
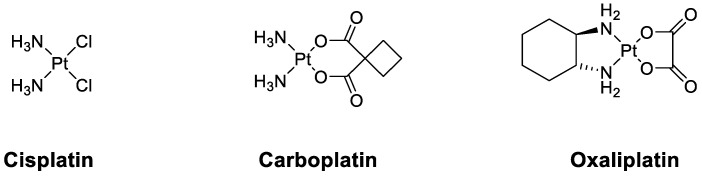
Structures of cisplatin, oxaliplatin, and carboplatin.

Metal complexes are currently playing a tremendous role in medicine. For example, the platinum complex cisplatin and its derivatives oxaliplatin and carboplatin (Figure 5) are employed in more than 50% of the treatment regimens for patients suffering from cancer!

Over the last years, our research group has focused its attention on the development of novel metal complexes as imaging and therapeutic agents against cancer and parasitic diseases [25,26,27,28,29,30]. During this talk, we will present our latest results, including in vivo results, on these topics.

### 2.10. Designing New Synthetic Concepts for Imparting Molecular Complexity with C-1 Sources (KL10)

**Vittorio Pace, Laura Ielo, Margherita Miele and Raffaele Senatore**

Department of Chemistry, University of Turin, Via P. Giuria 7-10125 Torino, Italy; vittorio.pace@unito.it

**Figure 6:**
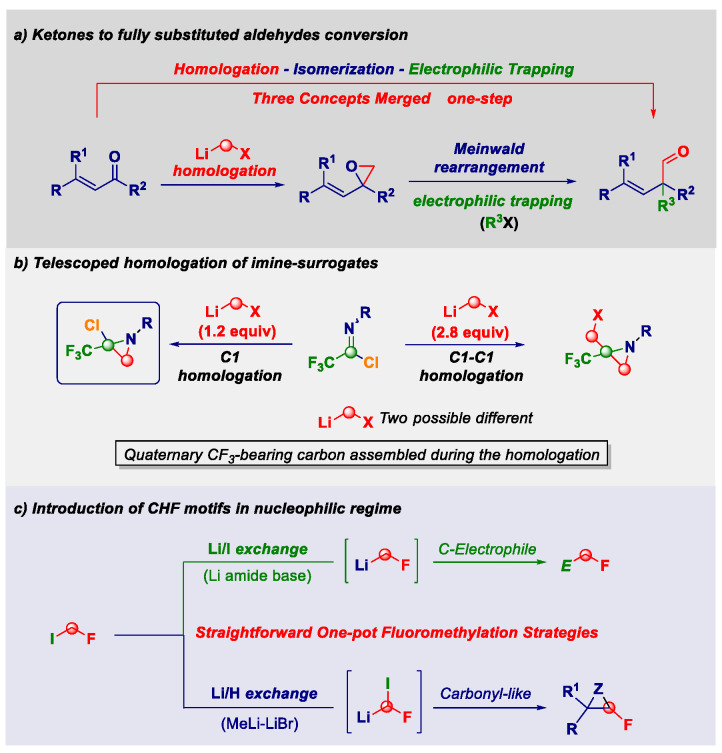
(**a**) Conversion of ketones to fully substituted aldehydes; (**b**) telescoped homologation of imine surrogates; (**c**) introduction of CHF motifs in nucleophilic regime.

The direct transfer of a reactive nucleophilic CH2X unit into an existing linkage enables the formal introduction of the moiety with a precisely defined degree of functionalization [31,32]. Upon the fine-tuning of the reaction conditions governing the transformation, the initial homologation event can serve as the manifold for triggering unusual rearrangement sequences leading to complex architectures through a unique synthetic operation. The direct/full chemoselective conversion of a ketone into the homologated all-carbon quaternary aldehyde (Figure 6a) [33] and the telescoped homologation of imine surrogates to quaternary aziridines (Figure 6b) [34] will illustrate these unprecedented concepts. Additionally, the one-step mono-fluoromethylation of carbon electrophiles with extremely labile fluoromethyllithium reagents will provide a novel entry to valuable fluorinated building-blocks without the need to use protecting elements for fluoro-containing carbanions (Figure 6c) [35,36,37]. Moreover, novel strategies for introducing the difluoromethyl group through the proper activation of commercially available TMSCHF_2_ with an alkoxide will be discussed [38,39].

## 3. Young Researcher Communications

### 3.1. All for One and One for All: Design and Synthesis of Molecules with Neuronal Differentiation Properties (YRC01)

**Mirjana Antonijevic ^1^, Isbaal Ramos ^2^, Maria Valcarcel ^2^, Patricia Villace ^2^, Patrick Dallemagne ^1^, Christophe Rochais ^1^**

^1^ Normandie Univ., UNICAEN, CERMN, 14000 Caen, France

^2^ Innoprot S.L, 48160 Derio, Bizkaia, Spain; mirjana.antonijevic@unicaen.fr

Numerous studies have been published about the implication of the neurotrophin tyrosine kinase receptor TrkB in the pathogenesis of several neurodegenerative conditions (Alzheimer’s disease, Parkinson’s disease, multiple sclerosis, and motor neuron disease) [40]. Brain-derived neurotrophic factor (BDNF) and neurotrophin-4/5 (NT-4/5) activate the TrkB receptor with high potency and specificity, promoting neuronal survival, differentiation, and synaptic function [41]. However, studies have shown that beside direct activation, TrkB receptor can be transactivated via GPCRs. Among all, it is proven that activation of 5-HT4 receptor, and transactivation of TrkB receptor, have a positive influence on neuronal differentiation (total dendritic length, number of primary dendrites, and branching index) [42]. Because of that, and based on the main structural characteristics of LM22A-4 [43], a known activator of the TrkB receptor, and RS67333 [44], a partial 5-HT4 receptor agonist, we have designed and synthesized a small set of compounds with potential dual activities, in order to not only prevent the death of the neurons, but also to induce neuronal differentiation in neurodegenerative disorders. During this communication, we will describe for the first time the new molecules with neuronal differentiation properties.

### 3.2. Synthesis and Biological Evaluation of Novel, Functionalized Bisindolylmaleimides (YRC02)

**Louise N. Cooney, Florence O. McCarthy**

School of Chemistry, Analytical and Biological Chemistry Research Facility, University College Cork, College Road, Cork, Ireland; 113352196@umail.ucc.ie

**Figure 7:**
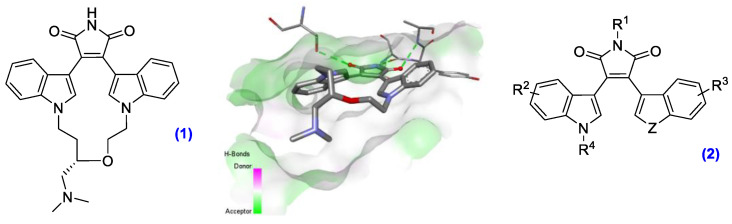
The bisindolylmaleimide ruboxistaurin **(1)** in the PDK1 kinase pocket (PDB: 1UU3) and the target compounds **(2)**.

Protein kinases are a class of regulatory enzymes and are ubiquitous within the human body. These enzymes are responsible for the modification of protein function through phosphorylation of target substrates, which have important downstream effects in intracellular signaling pathways [45]. Overexpression of particular kinases, including glycogen synthase kinase-3 (GSK-3), has been implicated in cancer and a number of neurological disease states including bipolar disorder and Alzheimer’s. This discovery of staurosporine propagated the design of 38 FDA approved kinase inhibitors, including the protein kinase C (PKC) inhibitor ruboxistaurin **(1)** (Figure 7) designed by Eli Lilly and Ro 318220, a nanomolar inhibitor of PKC and GSK-3β [46]. Crystal structures of this bisindolylmaleimide (BIM) framework identified that the maleimide headgroup (R^1^ = H) induces strong hydrogen bond interactions, with the hinge of amino acids locking it in place, and adopts the conformation represented in Figure 7 within the kinase pocket.

Structural modification and aryl replacement within the BIM frame is proven to improve kinase specificity and enhance anticancer activity [47]. This work describes the versatile synthetic route towards the novel BIM **(2)** pharmacophore by modifying the hydrogen-bonding headgroup R^1^ and incorporating different aryl units, as well as the assessment of steric probes R^4^. Molecular modelling was conducted on lead compounds to probe the influence of substituents on binding in the PKC and GSK-3β kinase active sites. A total of 125 novel drug candidates were prepared and tested using the National Cancer Institute 60-cell line screen, and nanomolar anticancer activity was exhibited.

### 3.3. C6-Modulation and Scaffold Hopping of Thienopyrimidinone Antiplasmodial Hit with Multi-Stage Activity (YRC03)

**Romain Mustière ^1^, Sébastien Hutter ^2^, Viviana Dell’Orco ^1^, Nadia Amanzougaghene ^3^, Shahin Tajeri ^3^, Céline Deraeve ^4^, Nadine Azas ^2^, Pierre Verhaeghe ^4^, Patrice Vanelle ^1^, Dominique Mazier ^3^, Nicolas Primas ^1^**

^1^ Aix Marseille Université, CNRS, ICR UMR 7273, PCR, Faculté de Pharmacie, Marseille, France

^2^ Aix Marseille Université, IHU Méditerranée Infection, UMR VITROME, Marseille, France

^3^ Sorbonne Université, CNRS/INSERM, CIMI, Paris, France

^4^ Université Paul Sabatier, CNRS UPR 8241, LCC, Toulouse, France; romain.mustiere@etu.univ-amu.fr

Malaria is a parasitic infection caused by *Plasmodium* that affected 229 million people and killed approximately 409,000 in 2019, according to the WHO [48]. The discovery of new antimalarial compounds is necessary to tackle the spread of artemisinin-resistant *Plasmodium falciparum* strains [49]. In this context, we identified M1, a lead compound belonging to the 2-aminothieno[3,2-*d*]pyrimidinones series [50], showing in vitro multi-stage antiplasmodial activity associated with low cytotoxicity. Moreover, M1 is active against quiescent artemisinin-resistant *P. falciparum* strain. Unfortunately, M1 is quickly metabolized by mouse liver microsome into inactive derivatives (t_1/2_ = 11 min), leading to activity loss in vivo in mouse models.

**Figure 8:**
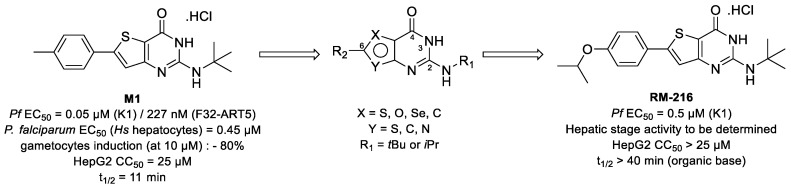
SAR studies conducted on the thieno[3,2-*d*]pyrimidine series.

To improve M1 microsomal stability and to complete the structure–activity relationship (SAR) studies, position 6 of the thieno[3,2-*d*]pyrimidine scaffold was modulated, and a scaffold hopping of the five-membered ring of the thienopyrimidine core was also investigated. All compounds were evaluated in vitro on the erythrocytic stage of *P. falciparum*. The best compounds were further assessed on the hepatic stage of *P. berghei,* and their in vitro metabolic stability was determined. Pharmacomodulations allowed us to discover new molecules with improved metabolic stability while limiting the loss of activity (Figure 8). Synthetic routes and biological results will be presented in the communication.

### 3.4. Benzimidazole Derivatives and Their Analogues as Myeloperoxidase Inhibitors (YRC04)

**Merve Saylam ^1^, Fadime Aydın Köse ^2^, Varol Pabuççuoğlu ^3^, Aysun Pabuççuoğlu ^4^**

^1^ Department of Pharmaceutical Chemistry, Faculty of Pharmacy, Izmir Katip Celebi University, Izmir, Turkey

^2^ Department of Biochemistry, Faculty of Pharmacy, Izmir Katip Celebi University, Izmir, Turkey

^3^ Department of Pharmaceutical Chemistry, Faculty of Pharmacy, Ege University, Izmir, Turkey

^4^ Department of Biochemistry, Faculty of Pharmacy, Ege University, Izmir, Turkey; merve.saylam@ikcu.edu.tr

Myeloperoxidase (MPO) enzyme is stored in neutrophils and plays a fundamental role in antimicrobial systems by generating hypochlorous acid (HOCl). Following phagocytosis of microorganisms, MPO is released into the phagolysosome and catalyzes the reaction between hydrogen peroxide (H_2_O_2_) and chloride ions (Cl^-^), which results in the production of highly oxidative HOCl. The antimicrobial activity of neutrophils is due to the damage potential of HOCl to vital biomolecules of the pathogen, such as DNA, RNA, proteins, lipids, and so on. Under certain circumstances, MPO may be released into extracellular fluids by continuing to generate HOCl. The formed HOCl there leads to tissue damage of the host, and these possible pathological changes might result in inflammatory diseases such as atherosclerosis, neurodegenerative diseases, renal injury, and so on [51,52,53]. Recently, the structure and function of MPO have been revealed in detail, knowledge which is essential for its development as an important therapeutic target.

According to the literature, it can be clearly seen that most of the compounds reported as MPO inhibitors have 5-membered fused rings, such as indole, indazole, and indazolone structures, beside the thiourea group [54,55,56,57]. Therefore, we designed and then synthesized benzimidazole-derived compounds and their bioisosteric analogues with MPO inhibitory potential. The activity of the compounds has been determined by taurine chloramine assay.

### 3.5. Synthesis and Chiral Resolution of Trans Configuration Anticancer β-Lactam Enantiomers (YRC05)

**Eavan McLoughlin, Niamh O’Boyle**

School of Pharmacy and Pharmaceutical Sciences, Trinity College, The University of Dublin, Trinity College Biomedical Sciences Institute, Pearse Street, Dublin 2; mclougea@tcd.ie

**Figure 9:**
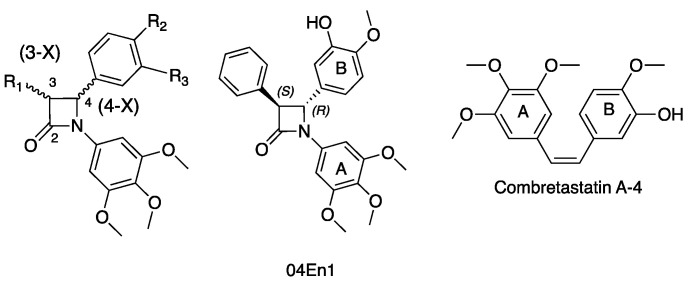
Stereochemistry of β-lactam enantiomers (**left**). **04En1** (3-*S*, 4*-R*) (**middle**) and CA-4 (**right**).

Combretastatin A-4 (CA-4) is a potent anticancer agent, which inhibits cancer cell proliferation and microtubule polymerization by binding at the colchicine-binding site of tubulin [58]. An extensive series of antiproliferative, tubulin-binding β-lactam compounds—the ‘combretazets’—have been reported by our group over the last decade with the aim of overcoming the undesirable in vivo *cis* to *trans* isomerization to CA-4′s inactive form (Figure 9) [59,60]. We have successfully optimized the Staudinger synthesis to optimize the yield of *trans* β-lactam isomers and successfully resolved the racemic mixture to afford optically pure enantiomers of a series of highly potent anti-cancer β-lactam derivatives.

Chiral high-performance liquid chromatography (HPLC) has demonstrated this resolution strategy as a means of isolating optically pure enantiomers in up to 90% enantiomeric excess. The (3-*S*, 4-*S*) and (3-*S*, 4-*R*) enantiomers demonstrate superior biological potency within the enantiomeric pair in the case of the 3-OH and 3-phenyl derivatives, respectively. Molecular modelling supports these findings, illustrating that the superior enantiomer maintains all crucial interactions at the colchicine-binding site (CBS). This is in contrast to the inferior (3-*R*, 4-*R*) and (3-*R*, 4*-S*) enantiomers, which dock inconsistently, with incorrect binding orientation. Preliminary findings demonstrate that the aforementioned (3-*S*, 4-*S*) and (3-*S*, 4-*R*) enantiomers exhibit potent anti-tubulin activity, while the inferior enantiomer does not. Compound **04En1** has demonstrated sub-nanomolar potency in a range of breast cancer cell lines inclusive of MCF-7 (IC_50_ 0.033 ± 0.013 nM), MDA-MB-231 (IC_50_ 0.679 ± 0.332 nM) and Hs578T(i)8 (IC_50_ 0.268 ± 0.058 nM), showing promising potential as a small-molecule chemotherapy agent for resistant and triple-negative breast cancers.

### 3.6. Peptide-Targeted Systems for Photodynamic Therapy (YRC06)

**Dong Wang ^1,2^, Sandy MacRobert ^3^, Charareh Pourzand ^1,2^, Ian Eggleston ^1,2^**

^1^ University of Bath, Department of Pharmacy and Pharmacology, Bath BA2 7AY, UK

^2^ Centre for Therapeutic Innovation, University of Bath, Bath BA2 7AY, UK

^3^ University College London, Division of Surgical and Interventional Sciences, London NW3 2PE, UK; dw816@bath.ac.uk

Photodynamic therapy (PDT) is a minimally invasive approach for the treatment of cancer and various other human disorders, based on the selective activation of photosensitizers (PSs) with light. At present, one of the most promising strategies for PDT and also fluorescence photodiagnosis (PDD) is to use 5-aminolevulinic acid (ALA) as a prodrug to increase intracellular levels of the endogenous PS, protoporphyrin IX (PpIX). Although ALA-PDT has been shown to be a very promising clinical approach, the physicochemical properties and chemical reactivity of ALA present some challenges. These may be addressed by incorporation of ALA units into a variety of prodrug systems [61], and we have previously shown that peptide-based prodrugs are an attractive way to improve the delivery of ALA, leading to enhanced PpIX accumulation and PDT effects [62]. In this study, we present a novel and easy strategy to assemble a prodrug system to enhance the delivery of ALA to specific cell-types using targeting with tumor-homing peptides. Our approach is based on a molecular core to which multiple ALA units (ALA dendron derivatives) are attached as the effector units, and with ALA itself connected by an ester bond. The core structure is also linked to a targeting peptide that is prepared by solid phase synthesis, with selective peptide attachment to the core being achieved via Cu-catalyzed click chemistry. This combines the concept of ALA dendrimers and ALA-peptide prodrugs [63]. As proof of concept of this particular approach, we have prepared systems containing a bombesin-derived peptide that allows selective targeting of the GRP receptor (GRPR), which is overexpressed in a variety of tumors. Targeted ALA delivery and PpIX production with these prodrugs in GRPR-expressing PC3 cells will be described.

### 3.7. SAR of Methylsulfanylpyridine-Based Combretastatin Analogues as Promising Antimitotic Agents (YRC07)

**Raquel Álvarez ^1^, Laura Aramburu ^1^, Consuelo Gajate ^2^, Faustino Mollinedo ^2^, Manuel Medarde ^1^, Rafael Peláez ^1^**

^1^ Department of Pharmaceutical Sciences, USAL, Campus Miguel Unamuno, E-37007, Salamanca, Spain

^2^ Department of Molecular Biomedicine, CIB, CSIC, E-28040, Madrid, Spain; raquelalvarez@usal.es

Antimitotic agents block cell division by interfering with the mitotic machinery. Ligands that bind to the colchicine domain in tubulin interfere with microtubule dynamics, polymers built up by the polymerization of α,β-tubulin dimers [64]. Despite the promising tubulin polymerization inhibition and cytotoxicity, combretastatin A-4 (CA-4) suffers from low aqueous solubility and configurational instability. The aim of this work is to enhance the overall polarity of the compounds piece by piece in a way that is tolerated by the highly hydrophobic site to avoid activity loss. We have combined three previous successful strategies of structural modification [65]: the first one consisting of A-ring replacement by substituted pyridines, the second one involving modifications on the B-ring that allow additional polarity enhancements, and finally, reduction of the two–atom combretastatin bridge to one-atom bridges, as in benzophenones, oximes, and 1,1-diarylethenes.

**Figure 10:**
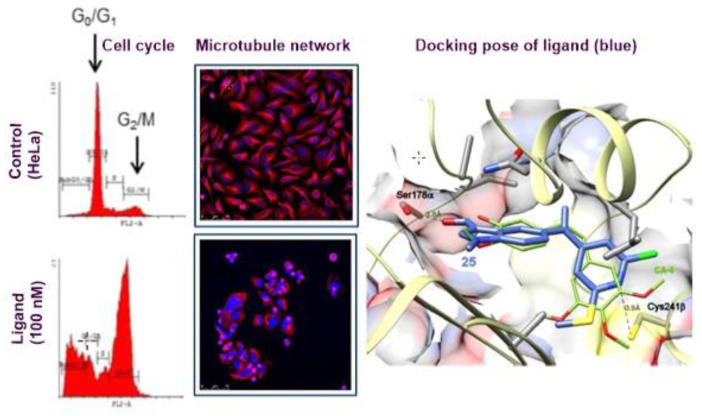
Left: Ligand effects on cell-cycle arrest and microtubule network. Right: Docking pose of one of methylsulfanylpyridine derivatives’ synthesized ligands (blue) superimposed with the X-ray pose of CA-4 (green).

The resulting molecules showed high antiproliferative activity against several cancer cell lines (i.e., HeLa, HT-29, and HL-60). The mechanism of action was assessed by tubulin inhibition polymerization experiments and immunofluorescence confocal microscopy. The observed microtubule disruption was accompanied by a cell cycle arrest at the G2/M phase, and subsequently, apoptotic cell death was confirmed by analyzing PARP cleavage, an early marker of apoptosis (Figure 10). Conformational and docking studies support the binding of the ligands at the colchicine site of tubulin.

Financial support: Consejería de Educación de la Junta de CyL and FEDER Funds (SA0116P20) and USAL (PIC2–2020-01).

### 3.8. Discovery and Binding Study of Herap2 Modulators Identified via Hts (YRC08)

**Pierre Sierocki, Laura Medve, Charlotte Fleau, Ronan Gealageas, Tuhina Khan, Rodolphe Vattinel, Valentin Guillaume, Melissa Rosell, Sandrine Warenghem, Florence Leroux, Catherine Piveteau, Benoît Deprez, Rebecca Deprez-Poulain**

Univ. Lille, Inserm, Institut Pasteur de Lille, U1177 -Drugs and Molecules for Living Systems, F-59000 Lille, France; pierre.sierocki@univ-lille.fr

**Figure 11:**
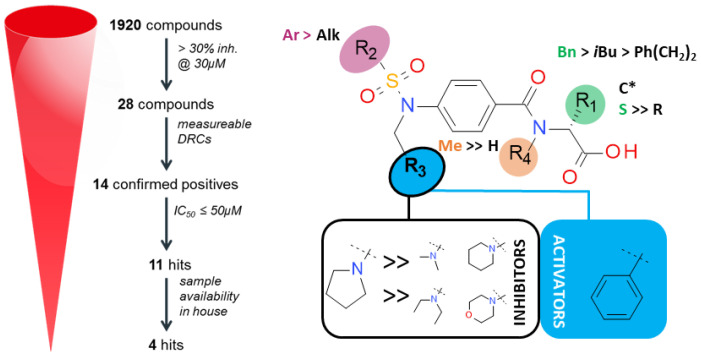
HTS campaign and SAR around a series of newly discovered ERAP2 modulators.

Endoplasmic reticulum aminopeptidase 2 (ERAP2) together with ERAP1 (50% homology) are responsible for antigenic peptides processing inside the ER and further addressing to MHC-I. Recently, ERAP2 has emerged as a promising pharmacological target through the validation of its role in several pathologies, among which are autoinflammatory diseases and cancer [66,67], while some selective ERAP1 modulators have already been disclosed. Herein, we would like to report on the discovery and the unprecedented binding mode of a series of modulators recently identified by the team using an HTS approach (Figure 11) [68].

## 4. Posters

### 4.1. Theoretical Insights into the Anti-SARS-CoV-2 Activity of Chloroquine and Its Analogs and In Silico Screening of Main Protease Inhibitors (P01)

**Achutha Anil, Pushpa V L, Suchitra Surendran**

Post-graduate and Research Department of Chemistry, DST-FIST Supported Department, Sree Narayana College, Kollam, Affiliated to University of Kerala-691001, India; achutha.anil@gmail.com

**Figure 12:**
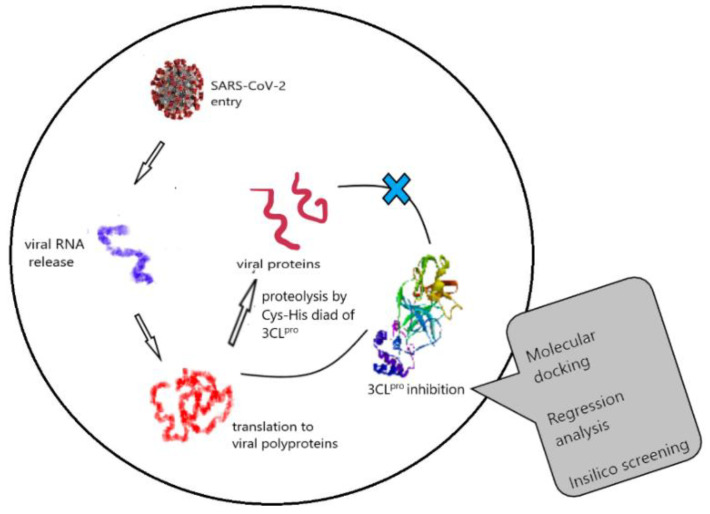
Design of inhibitors of main protease using in silico tools.

Coronavirus disease (COVID-19) is a dangerous disease rapidly spreading all over the world today. Currently, there are no treatment options for it. Drug repurposing studies explored the potency of antimalarial drugs, chloroquine and hydroxychloroquine, against the SARS-CoV-2 virus. These drugs can inhibit the viral protease, called chymotrypsin-like cysteine protease, also known as main protease (3CL^pro^); hence, we studied the binding efficiencies of 4-aminoquinoline and 8-aminoquinoline analogs of chloroquine (Figure 12). Six compounds furnished better binding energies than chloroquine and hydroxychloroquine. The interactions with the active site residues, especially with Cys145 and His41, which are involved in catalytic diad for proteolysis, make these compounds potent main protease inhibitors. A regression model correlating binding energy and the molecular descriptors for chloroquine analogs was generated with R^2^ = 0.9039 and Q^2^= 0.8848. This model was used to screen new analogs of primaquine and molecules from the Asinex compound library. The docking and regression analysis showed these analogs to be more potent inhibitors of 3CLpro than hydroxychloroquine and primaquine. The molecular dynamic simulations of the hits were carried out to determine the binding stabilities. Finally, we propose four compounds which show drug likeness towards SARS-CoV-2 that can be further validated through in vitro and in vivo studies [69].

### 4.2. Novel Conjugation Approach to Attain Immunoconjugates of Monoclonal Antibodies (P02)

**Bayan Alkhawaja ^1,2^, Andrew G. Watts ^2^, Andreas Michael ^2^, Rebecca Martin ^2^**

^1^ Faculty of Pharmacy and Medical Sciences, The University of Petra, Amman, Jordan

^2^ Department of Pharmacy and Pharmacology, University of Bath, Bath BA2 7AY, United Kingdom; Bayan.Alkhawaja@uop.edu.jo

Over the last two decades, monoclonal antibodies (mAbs) and mAbs-based bio-therapeutics have attained considerable attention as a targeted treatment in the oncology field. Both antibody drug conjugates and bispecific antibodies have been established to empower the widespread benefits experienced with mAbs [70]. Bioconjugation chemistry has enabled the recent huge growth of these mAbs-based bio-therapeutics. The current conjugation methods are based on conventional chemistries to label native amino acid residues. However, the current bioconjugation chemistries have a wide range of limitations [71].

**Figure 13:**
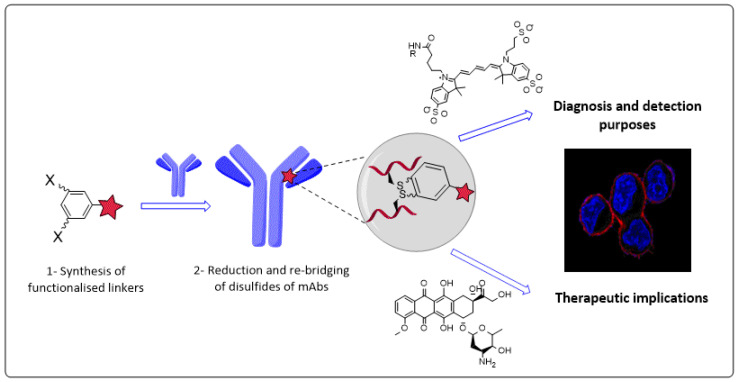
Our approach to obtain labelled conjugates of antibodies.

The primary focus of our research is developing novel affordable chemistries to introduce covalent functionalities to mAbs, whilst maintaining the structural integrity and stability, hence tackling the bespoke limitations experienced with current conjugation methods (Figure 13).

Herein, the developed bioconjugation platforms are based on bis-reactive aryls proposed to rebridge the disulfide bonds of mAbs. The widespread advantages of these platforms could facilitate the construction of ground-breaking therapeutics, including labelled as well as armed immunoconjugates of monoclonal antibodies.

### 4.3. Inhibitors and Activators of Pyruvate Kinase Muscle Isozyme Splice Variant 2 (Pkm2): An Overview (P03)

**Sahil Arora, Raj Kumar**

Laboratory for Drug Design and Synthesis, Department of Pharmaceutical Sciences and Natural Products, Central University of Punjab, Ghudda, 151401 Bathinda, India; deepsahil7999@gmail.com

**Figure 14:**
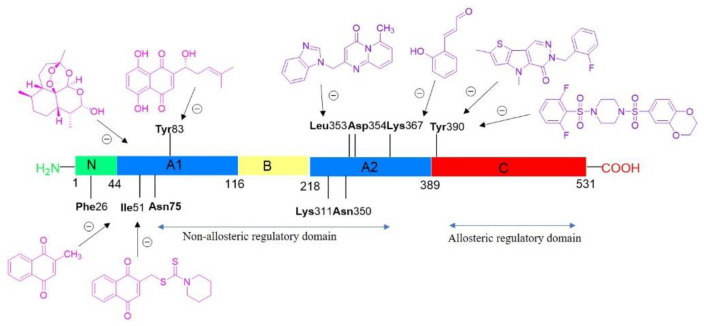
Some of the PKM2 activators and inhibitors binding PKM2 domains.

Pyruvate kinase M2 (PKM2) plays a major role in altered metabolic regulation and acts as a rate-limiting enzyme in the last step of glycolysis, which catalyzes the conversion of phosphoenolpyruvate to pyruvate and generates ATP from ADP. PKM2 actively participates in the production of high lactate levels in aerobic conditions and PKM2 is mainly expressed in leukemia and other cancers such as lung, pancreatic, gastrointestinal, ovarian cancer, and so on, via PKM2 upregulation. Literature findings reveal many PKM2 inhibitors and activators (Figure 14). PKM2 inhibitor of the natural ligand shikonin, which had been reported as a specific PKM2 inhibitor, limits its use, as it targets both PKM2 and mitochondria, not revealing an understanding of the role of PKM2 in cancer metabolism. Further, there is no FDA-approved PKM2 inhibitor, and there is no consensus approach for the design and synthesis of effective PKM2 inhibitors. Due to the central regulatory role of PKM2 in many cancers, there is an urgent need for more effective PKM2 inhibitors. Besides the therapeutic benefit of PKM2 inhibition, it is better to understand the function of PKM2 in normal and cancer cells [72,73,74].

### 4.4. Self-Assemblies of Azacitidine Prodrug: An Innovative Therapy against Myelodysplastic Syndromes (P04)

**Milad Baroud ^1^, Elise Lepeltier ^1^, Sylvain Thepot ^2,3,4^, Yolla El-Makhour ^5^, Olivier Duval ^1,2^**

^1^ Micro et Nanomédecines Translationnelles, MINT, UNIV Angers, UMR INSERM 1066, UMR CNRS 6021, Angers, France

^2^ University Hospital of Angers, Hematology, 49933 Angers, France

^3^ Université d’Angers, Inserm, CRCINA, 49000 Angers

^4^ Fédération Hospitalo-Universitaire ‘Grand Ouest Against Leukemia’ (FHU GOAL), France

^5^ Environmental Health Research Lab (EHRL), Faculty of Sciences V, Lebanese University, Nabatieh, Lebanon; milad.baroud@etud.univ-angers.fr

A cytidine analogue and hypomethylating agent, 5-Azacitidine is one of the main drugs being used for the treatment of myelodysplastic syndromes [75]. However, after administration, it exhibits several limitations, including restricted diffusion and cellular internalization due to its hydrophilicity, and rapid enzymatic degradation by adenosine deaminase. The aim of this study was to improve the drug diffusion and protect it from metabolic degradation via the formulation of an amphiphilic prodrug and its self-assembly into a nanoparticle. The alcohol groups of azacitidine were first protected using TBDMS to inhibit secondary conjugations, followed by the coupling of an unsaturated fatty acid to the amine group, and subsequently, deprotection was accomplished using TBAF, thus yielding an amphiphilic prodrug [76]. Next, the obtained prodrug was solubilized in acetone and mixed with water at different ratios to obtain self-assemblies by nanoprecipitation, thus protecting the active molecule from enzymatic degradation. This prodrug should be cleaved by cathepsin B, which is overexpressed in cancerous cells, therefore increasing the specificity of the drug [77,78]. Furthermore, its amphiphilic nature should aid diffusion. This strategy would allow protection while increasing azacitidine’s specificity and bioavailability.

### 4.5. Genistein as Drug of Choice for Sars Cov2 Infection Using Drug-Gene Network (P05)

**Anamika Basu**

Department of Biochemistry, Gurudas College, Kolkata, India; basuanamikaami@gmail.com

**Figure 15:**
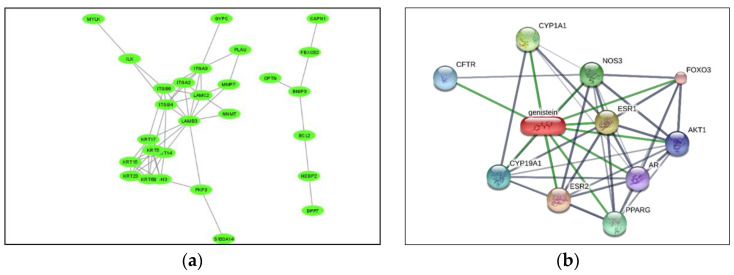
(**a**) Differentially expressed gene network. (**b**) Drug–gene network for genistein.

A system-pharmacology-based drug–gene network can combine two types of interactions. The first one highlights the interactome with human host gene co-expression network for a SARS-CoV2-virus-infected individual. In the case of the second type of interaction, a drug–gene network can be constructed with the differentially expressed genes obtained from the first network. From this system-pharmacology-based approach, the drug of interest for SARS CoV2 can be identified. From the RNA expression dataset, comparing a normal and COVID-19-infected patient, a gene expression network has been constructed, with the help of network biology software (as shown in Figure 15a). By using the clustering method, forty-four genes are identified in cluster 1 among four clusters with differentially expressed genes. From the gene annotation study for these genes, the importance of hemidesmosome complex in cell-to-cell transmission of SARS CoV2 has been identified (unpublished results).

For a ligand-based drug–gene network, the above-mentioned forty-four genes and twelve chemical compounds are selected using ToppGeneFun software [79]. Among them, only the natural product genistein is selected as a drug for the treatment of COVID-19 infection. Genistein is a phytoestrogen and belongs to the category of isoflavones. The gene network with genistein drawn with the STITCH database is shown in Figure 15b [80]. The effect of genistein on hemidesmosome complex is studied with in silico techniques.

### 4.6. Design and Study of Potential FabZ Inhibitors as Antimicrobial Drugs (P06)

**Laurie Bibens ^1^, Jean-Paul Becker ^1^, Nadine Lemaitre ^1^, Céline Damiani ^1^, Nicolas Taudon ^2^, Alexandra Dassonville-Klimpt ^1^, Pascal Sonnet ^1^**

^1^ AGIR, UR 4294, Université de Picardie Jules Verne, Amiens, France

^2^ Unité de Développements Analytiques et Bioanalyse, IRBA, Brétigny-sur-Orge, France; laurie.bibens@u-picardie.fr

To date, antimicrobial resistance has been one of the biggest public health challenges. Multi-resistance is particularly worrying in Gram-negative bacteria isolated from nosocomial infections such as *P. aeruginosa*, *E. coli*, or *K. pneumoniae*. Each year, at least 700,000 people die from antibiotic-resistant infections worldwide [81]. The decrease in the effectiveness of antimalarial treatments also gives rise for concern. Indeed, *P. falciparum*, the most virulent *Plasmodium* species, is responsible for most of the 409,000 deaths reported in 2019 [48].

Therefore, it is urgent to propose novel treatments with original and selective antimicrobial modes of action. Lipids are essential in maintaining bacterial membrane integrity. Their biosynthesis involves both fatty acid synthase-I (FAS-I) and fatty acid synthase-II (FAS-II). FAS-II is uniquely found in bacteria, plants, and apicomplexan parasites, such as *Plasmodium*. Furthermore, the FAS-II enzymes have a high level of conservation in the microbial pathogens. Among these, FabZ, a β-hydroxyacyl-acyl carrier protein (ACP) dehydratase, plays a pivotal role in the FAS II. This suitable yet little-explored enzyme represents a promising target to design broad-spectrum antimicrobials with limited side effects and offers minimum chances of cross-resistance with existing drugs targeting other pathways.

Very few FabZ inhibitors have been described, while several FabZ 3D structures from different organisms such as *P. aeruginosa*, *P. falciparum*, and *H. pylori* have been reported [82,83]. Among known FabZ inhibitors, the NAS91 family, with a quinoline core, inhibits PfFabZ with IC_50_ in a micromolar range. Additionally, co-crystal NAS91 family-PfFabZ complex structures are described in the Protein Data Bank (PDB) (3AZA, 3AZ9, 3AZB). Based on these data, we have started a FabZ-based drug design study to propose new quinoline structures. The in silico study, synthesis of some new quinolines, and first biological results will be shared.

### 4.7. Determining Particle Impurity Distributions in Pharmaceutical Solids (P07)

**Timothy Bourke ^1^, Humphrey Moynihan ^1^, Renato Chiarella ^2^**

^1^ School of Chemistry, University College Cork, Ireland, T12 ND89

^2^ Alkermes, Inc., 852 Winter Street, Waltham, MA 02451-1420, USA; timothy.bourke@umail.ucc.ie

**Figure 16:**
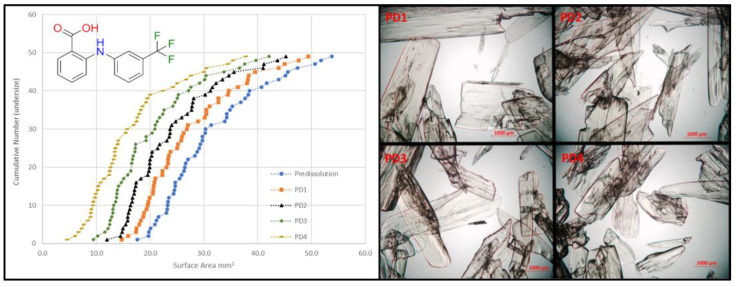
Size distributions (**left**) and microscope images (**right**) over a series of stepwise dissolutions.

Purity is a critical attribute of medicinal compounds. In pharmaceutical manufacturing, the presence of unwanted impurities is a significant problem in batches of drug products, many of which are difficult to remove by standard purification methods such as washing or recrystallization. [84]. This project is focused on developing reliable methods of analyzing the distribution of impurities incorporated within crystals of pharmaceutical products for use in industry, with the aim of better determination of effective purification methods. Five compounds were designed and synthesized as structurally similar additives to an API host, flufenamic acid (FFA), intended to mimic such impurities by forming substitutional solid solutions resistant to purification (Figure 16).

Samples of FFA were crystallized after having been doped with low levels of additive to produce such systems. The behavior of the doped systems was investigated, and the solid-state relationships characterized by HPLC, PXRD, TGA, and DSC analysis, including the construction of phase diagrams. A stepwise dissolution method was developed that allowed for the controllable dissolution of a single crystal or batch of crystal particles across multiple dissolution stages in order to map the distribution of one or more additives/impurities within the crystal(s) on a surface-to-center basis (Figure 1). This method was successfully applied to systems with a variety of additive levels, additive types, and crystal morphologies. An alternative method of determining impurity distributions within acicular crystals was also developed. Employing these methods in an industry setting would allow a researcher to gain insight into the distribution of impurities within a batch of drug product, and as such inform a strategy for purification.

### 4.8. The Synthesis of Enantiopure DSA Analogues for the Treatment of Cancer (P08)

**Lily Cassidy, Andrew Beekman, Mark Searcey**

School of Pharmacy, University of East Anglia, Norwich Research Park, NR47 TJ Norwich, UK; lily.cassidy@uea.ac.uk

Cancer is the second leading cause of death in the world, with more than one in two people being diagnosed within their lifetime [85]. Cancer can be treated through different means, including chemotherapy, and the choice of drug depends on the type of cancer and the extent of its spread.

The duocarmycins are natural compounds that are highly potent DNA-alkylating agents. They bind to the minor groove in AT-rich regions of DNA and are among the most potent cytotoxic compounds known, offering a great potential for cancer treatment. However, high toxicity and low selectivity mean there has been limited progress of duocarmycins as chemotherapeutic agents. One approach to achieve selective cytotoxicity is with antibody drug conjugates, or ADCs. Here, the cytotoxic drug is bound by a linker to the antibody, and in doing this, the payload can be targeted directly to the tumors. A duocarmycin-based ADC called SYD985 is in phase III clinical trials for the treatment of HER-2 metastatic breast cancer [86]. This shows an exciting new field to be explored, and this project will look at preparing a dimeric payload that could potentially be used as a warhead for an ADC.

The duocarmycin family consists of different alkylating subunits, including the duocarmycin SA unit, DSA, one of the most potent subunits which is of great interest in the design of analogues [87]. This project follows work previously done by Boger, where yatakemycin and DSA were synthesized stereoselectively. Enantiopurity is important as it affects how the compounds will bind to DNA and therefore the potency. Once the DNA-alkylating unit has been synthesized in a stereoselective fashion and with an appropriate protection strategy, analogues can be made using solid phase synthesis techniques already established within the research group. This SPS method allows a library of analogues to be made and studied, with a focus on making dimers. Duocarmycin payloads in ADCs have shown promise as dimers with both dimeric seco-CBI payloads and PBD dimers in clinical trials [88]. Using our approach, we will be able to “tune” the physicochemical characteristics of the dimers by varying amino-acid-based linker structures.

### 4.9. Novel Synthesis of Pyrroloquinoxalines Using Sulfone Radicals (P09)

**Amy Chave, Bhaven Patel, Daniel Sykes**

School of Human Sciences, London Metropolitan University, 166–220 Holloway Road, N7 8DB London, UK; a.chave1@londonmet.ac.uk

**Figure 17:**
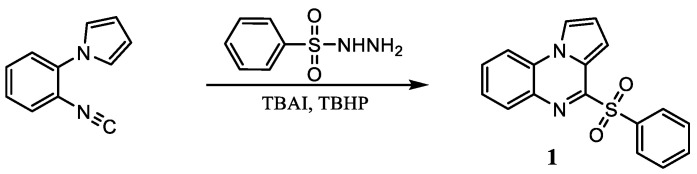
Radical isocyanide cyclization with benzene sulfonyl hydrazide in the presence of TBAI and TBHP.

Marinoquinolines (Mq) are a group of natural products that share a pyrroloquinoline core and are derived from marine bacteria. At the time of writing, eleven Mq have been isolated (Figure 1). Mq and derivatives have been found to be effective acetylcholinesterase inhibitors (IC_50_ = 4.9 μM) [89] and *Plasmodium* spp. inhibitors (IC_50_ < 1 μM) [90]. Pyrroloquinoixalines are tricyclic Mq derivatives, some of which have been demonstrated within the group to be acetylcholinesterase inhibitors (IC_50_ = 2 μM). In terms of synthesis, there are several viable routes to tricyclic heterocycles, with reported examples including a Pictet–Spengler reaction [91] and Ullman coupling of pyrroles with nitroarenes [92]. Radical chemistry represents an attractive alternative thanks to its mild conditions and lack of necessity to protect functional groups [93]. However, several radical syntheses use transition metal catalysts that are non-trivial to eliminate from reaction mixtures and may be toxic. A metal-free alkyne cyclization using sulfone radicals generated in the presence of tetrabutylammonium iodide (TBAI) and *t*-butyl hydroperoxide (TBHP) has been previously reported [94]. Here, we present a novel metal-free radical isocyanide cyclization with sulfone radicals in 12% yield (Figure 17). The benzene sulfonyl group is a good leaving group, making compound **1** a suitable precursor for synthesis of other substituted pyrroloquinoxalines by substitution under basic conditions.

### 4.10. Synthesis of Potential Antitumor Agents Targeting DNA G-4 and Kinases (P10)

**Italo Cirone, Elias Maccioni**

Dept of Life and Environmental Sciences, University of Cagliari, Cagliari (CA), Italy; italo.cirone93@gmail.com

**Figure 18:**
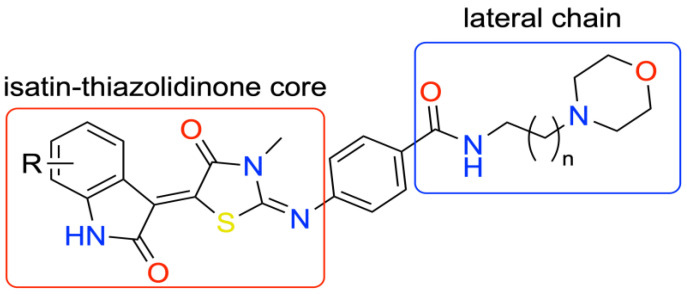
General structure of proposed dual-targeting hit compounds.

Due to the low selectivity and severe side effects of conventional anticancer therapies, research is currently focusing on new small molecules targeting specific cancer cell mechanisms, which can eventually lead to new drugs with a good therapeutic index and decreased side effects. Two of the most studied targets for this kind of approach are the protein kinase family and DNA G-Quadruplex. The development of dual-targeting small molecules is highly attractive because single-target drugs, in order to be effective and to avoid the appearance of resistance, need to be used in a cocktail. This could lead to compounded side effects, whereas the use of a single molecule acting on all the targets could reduce the risk of severe side effects [95]. In light of the above, two currently marketed kinase inhibitors, Sunitinib and Nintedanib, were taken as a starting point to design eight hit compounds, all carrying an isatin moiety. Isatin is a recurring motif in a variety of natural active compounds and, considering its low toxicity, is considered a privileged scaffold in drug design. These eight hit compounds feature a thiazolidine-indolinone core carrying, at the 2 position of the thiazolidinone ring, a morpholinic lateral chain, to enhance solubility in water (Figure 18).

These molecules were synthesized with a straightforward five-step synthetic route in quantitative yields. Subsequently, a prediction of their drug-like properties was performed using Swiss ADME. All compounds were reported to have a good lipophilic/hydrophilic balance. These hit compounds are promising candidates, and their properties should be further investigated through the relevant biological assays.

### 4.11. Novel Bioactive Benzimidazoles (P11)

**Chiara Maria Costanzo ^1^, Suzy Claire Moody ^2^, E. Joel Loveridge ^1^**

^1^ Dept of Chemistry, Swansea University, Singleton Park, Swansea, SA2 8PP, Wales

^2^ Dept of Pharmacy and Chemistry, Kingston University, Kingston, KT1 2EE, United Kingdom; 993910@swansea.ac.uk

**Figure 19:**
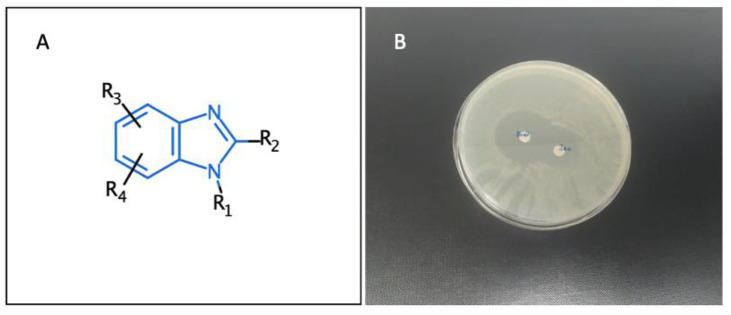
(**A**) Benzimidazole general structure; (**B**) synergistic effect of a derivative synthesized in this work and tested against E. coli through disk diffusion assay.

Benzimidazole plays a key role in medicinal chemistry. Its derivatives have been evidenced to have multiple pharmacological properties, such as antiviral, anticancer, anti-inflammatory, antioxidant, and antimicrobial [96,97,98,99]. This work investigates the reactivity of the benzimidazole core in order to fully explore the effect of substituents on its different extensible sites (Figure 19A) and produce a structure–activity relationship.

A library of substituted benzimidazoles has been synthesized, from substituted o-phenylenediamines and alkyl carboxylic acids or aromatic aldehydes. Furthermore, several of the compounds synthesized have shown promising bioactivity. Both of these aspects of the work will be described. This work will contribute to the discovery of new benzimidazole-based anti-infective agents.

### 4.12. Photocatalytic α-C–H Functionalization of Unprotected Primary Alkylamines (P12)

**Alexander J. Cresswell**

Department of Chemistry, University of Bath, Claverton Down, Bath, BA2 7AY, U.K.; a.j.cresswell@bath.ac.uk

**Figure 20:**
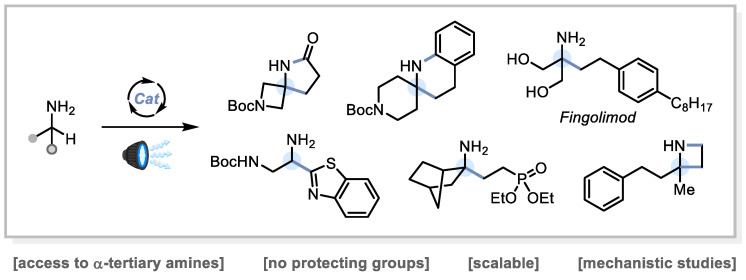
Photocatalytic α-C–H functionalization of primary aliphatic amines.

My group has recently found that primary alkylamines without *N*-protection can be employed directly in photoredox catalysis to form new C–C bonds α- to nitrogen, using a variety of radicophiles as coupling partners [100,101]. This is a key advance for amine synthesis, providing a highly simplifying disconnection for α-tertiary amines and saturated azacycles, including spirocycles. These compounds are of significant interest in drug design. Our strategy uses an organic photocatalyst in combination with a hydrogen atom transfer (HAT) catalyst, and we have so far applied this methodology to the direct synthesis of C-alkylated primary amines, γ-lactams, γ-aryl amines, tetrahydroquinolines, and α-(benzo)thiazolyl amines (Figure 20). Most of these compounds are cumbersome to access using the current state of the art. The scalability of the chemistry has been demonstrated in continuous flow (on decagram scale), and detailed kinetic and photophysical studies have provided astounding detail on the intricate workings of the reactions, including photocatalyst deactivation processes. We have recently applied this methodology to a single-step synthesis of the blockbuster drug fingolimod, and we are now applying it further towards the synthesis of other medicinally or biologically relevant amine targets.

### 4.13. New Orthogonal Decoration of 4-Amino-pyrido[2,3-d]pyrimidin-7(8H)-ones (P13)

**Claudi de Rocafiguera, Iñaki Galve, Raül Ondoño, Raimon Puig de la Bellacasa, José I. Borrell**

Grup de Química Farmacèutica, Organic and Pharmaceutical Chemistry Department, Institut Químic de Sarrià, Universitat Ramon Llull, Via Augusta, 390, E-08017 Barcelona, Spain; crocafiguerav@iqs.url.edu

**Figure 21:**
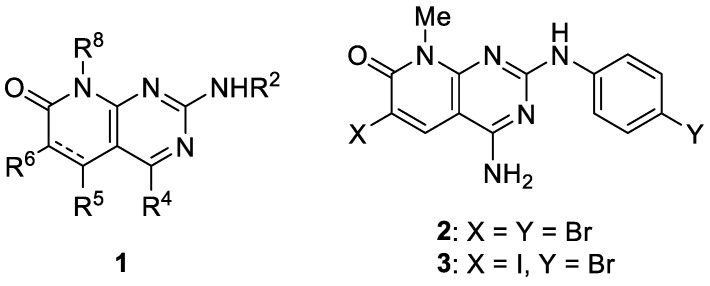
Structure of pyrido[2,3-*d*]pyrimidin-7(*8H*)-one (**1**) and the common starting reagent.

Pyrido[2,3-*d*]pyrimidin-7(8*H*)-ones are bicyclic heterocyclic compounds for which very interesting inhibitory activities have been described in the field of protein kinase inhibitors. Our group has described in the past years several straightforward strategies for the synthesis of 4-amino and 4-oxo substituted pyrido[2,3-*d*]pyrimidin-7(8*H*)-ones **1** (R^4^ = NH_2_/OH) (Figure 21) with up to five diversity centers and two possible degrees of unsaturation in the pyridone ring. Consequently, an adequate decoration of structures **1** has allowed us to describe compounds with nM activities as breakpoint cluster region protein (BCR) kinase inhibitors for B lymphoid malignancies, discoidin-domain-containing receptor (DDR2) inhibitors for treatment of lung cancer, hepatitis C virus (HCV) inhibitors, and others. A drawback of our synthetic methodologies is the fact that a de novo synthesis is needed each time a new pyrido[2,3-*d*]pyrimidin-7(8H)-one with a different set of substituents in R^6^ and at the para position of the phenylamino substituent at position C^2^ is required.

In this context, our group has developed a methodology to obtain pyrido[2,3-*d*]pyrimidin-7(*8H*)-ones easily amenable to decoration by using a dihalogenation methodology with bromo and iodo reagents to render the dibromo **2** or 2-((*p*-bromophenyl)amino)-6-iodo **3** compounds as a common starting reagent. Then, once the two dihalo-substituted compounds were obtained, we carried out a proof of concept of the orthogonality of the halogens present in both compounds using a Suzuki–Miyaura coupling reaction protocol. The reactivity differentiation of the 6-iodo compound **3** allowed the regiospecific cross-coupling at position C6 with a wide range of arylboronic acids. The iodine and bromine atoms present in compound **3** can be sequentially substituted using Suzuki, Ullman, and other protocols to achieve potentially active tyrosine kinase inhibitors. Such orthogonal decoration of compound **3** allows a rapid and convenient approach to pyrido[2,3-*d*]pyrimidin-7(*8H*)-ones without needing a de novo synthesis from an α,β-unsaturated ester bearing the aryl substituent for each combination of substituents [102].

### 4.14. Tyrosine Selective Bioconjugation Using An Electro-Oxidative Methodology for Biomolecules Labeling (P14)

**Sébastien Depienne ^1^, Dimitri Alvarez-Dorta ^1^, Mikael Croyal ^2^, Estelle Lebegue ^1^, Christine Thobie-Gautier ^1^, Mohammed Boujtita ^1^, David Deniaud ^1^, Sébastien G. Gouin ^1^**

^1^ CEISAM Laboratory UMR 6230, Nantes University, F-44000 Nantes, France

^2^ CHU Nantes, SFR Santé, Inserm UMS 016, CNRS UMS 3556, Nantes University, F-44000 Nantes, France; sebastien.depienne@univ-nantes.fr

**Figure 22:**
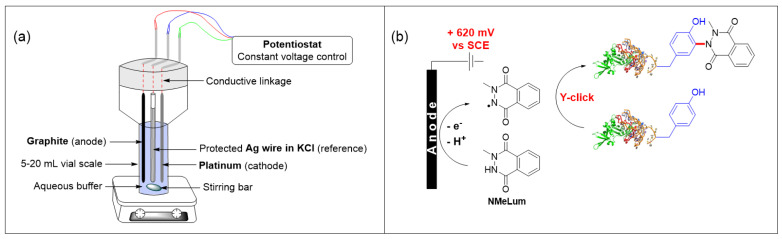
(**a**) Three-electrodes system connected to a potentiostat used for constant voltage experiments. (**b**) NMeLum radicals are in situ progressively generated at the anode surface and further react with exposed Y from biomolecules.

Methodologies for simple and clean protein engineering are extensively explored for the development of new protein–drug conjugates, targeted medical imaging agents, or multifunctional biological tools. In particular, homogeneous protein labeling remains a distinctive challenge to overcome uncontrolled drug-loading and pharmacodynamics limitations of current conjugates. For this purpose, site-selective labeling of the less abundant surface-exposed tyrosines (Y) represents a constructive strategy. We recently developed the first electrochemical bioconjugation protocol, termed eY-click, to tag Y with 4-phenylurazole anchor under mild and traceless oxidative conditions [103]. The eY-click methodology was here implemented to a range of labeling reagents, highlighting 2-methyl-2,3-dihydropthalazine-1,4-dione (NMeLum) as a highly efficient and chemoselective Y-modifier under single-electron anodic oxidation. NMeLum was electro-oxidized in situ in pure aqueous buffer without affecting protein integrity. The generated radicals react readily with the phenol moiety of exposed tyrosines to obtain labeled biomolecules (Figure 22). The methodology proved efficient at low protein concentration, with biologically relevant targets (enzymes, antibodies), and with an azido-armed NMeLum anchor being further functionalized with strained cyclooctynes probes by SPAAC.

### 4.15. Modification of Carbon Surface to Enhance Removal of Cadmium from Aqueous Solution (P15)

**Rimene Dhahri ^1^, Younes Moussaoui ^2^**

^1^ Dept Faculty of Sciences of Gafsa, University of Gafsa, Tunisia.

^2^ Organic Chemistry Laboratory, Faculty of Sciences of Sfax, University of Sfax, Tunisia; dhahririmene@gmail.com

In this study, low-cost activated carbon was prepared from prickly pear seed cake biomass after bio-oil extraction, for the removal of cadmium ions from aqueous solution. The obtained adsorbent was treated with hydrogen peroxide H_2_O_2_ in order to introduce oxygen surface complexes and to evaluate the adsorption performance after functional surface modification. The effect of the oxidizing treatments on activated carbon was examined by several techniques, including nitrogen adsorption and SEM. Batch adsorption experiments were carried out to evaluate the effect of process parameters (pH solution, adsorbent dosage, contact time, and initial metal ions concentration) on the metal ions removal.

Langmuir and Freundlich adsorption models were employed to provide a description of the equilibrium isotherm. Furthermore, pseudo-first-order and pseudo-second-order kinetic models were conducted to investigate the mechanism of Cd(II) adsorption by the obtained adsorbent. The results show that the adsorption of Cd(II) follows the pseudo-second-order model kinetics. In addition, the adsorption process was correlated with the Langmuir model.

### 4.16. From Innovation to the Market: Adding Value to the Compounds from Academic Research and Teaching (P16)

**Divneet Kaur ^1^, Nicholas Bennett ^1^, Trevor Farren ^1^, Jody Ali ^2^, Steve Brough ^2^**

^1^ School of Chemistry, Business Partnership Unit, University of Nottingham, University Park, Nottingham NG7 2RD, UK

^2^ Key Organics, Camelford, Cornwall PL32 9RA, UK; divneet.kaur1@nottingham.ac.uk

The university’s synthetic chemistry researchers generate a significant quantity of novel and unique chemical compounds in the course of their research. These compounds include intermediates, reagents, fragments, ligands, ‘drug-like’ molecules, inorganic materials, and polymers that are often unavailable on the global market. However, on completion of the projects, many of these compounds have no further use and end up being stored and eventually disposed of.

**Figure 23:**
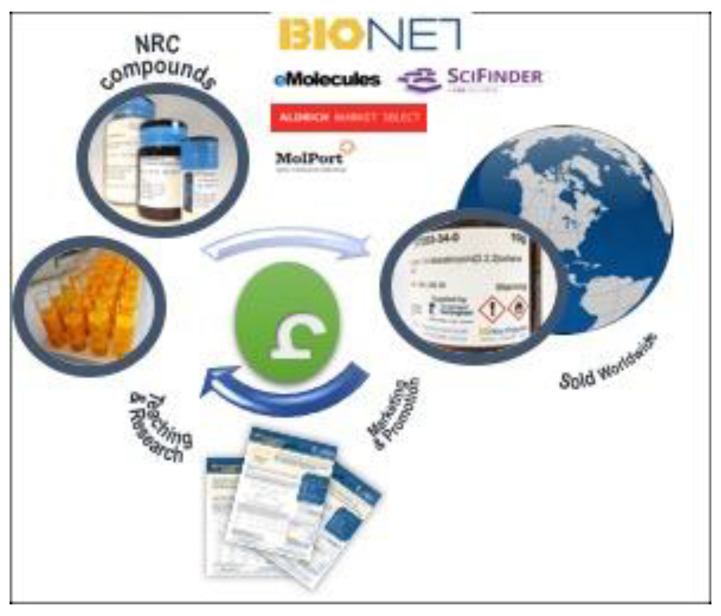
Nottingham Research Chemicals.

To maximize the value of these chemicals, a unique initiative was established in the School of Chemistry, UoN, called Nottingham Research Chemicals (NRC). This pioneering project allows the introduction of chemicals from research and teaching to the market via collaboration with our industrial partner, Key Organics Ltd. Since mid-2015, the NRC project has introduced >150 compounds to the market and continues to grow its portfolio (Figure 23). Additional advantage comes with promotional materials which highlight the state-of-the-art research ongoing at the University of Nottingham.

### 4.17. Antibiofilm Properties of Indolo[2,3-b]quinoline Derivatives (P17)

**Stéphane Gérard ^1^, Ludovic Doudet ^1^, Emilie Charpentier ^2^, Ingrid Allart-Simon ^1^, Sophie Gangloff ^2^, Fany Reffuveille ^2^**

^1^ ICMR UMR CNRS 7312, UFR de Pharmacie, Laboratoire de Chimie Thérapeutique, Université de Reims Champagne-Ardenne, SFR Cap Santé, France

^2^ EA 4691 BIOS « Biomatériaux & inflammation en site osseux », Université de Reims Champagne-Ardenne; stephane.gerard@univ-reims.fr

**Figure 24:**
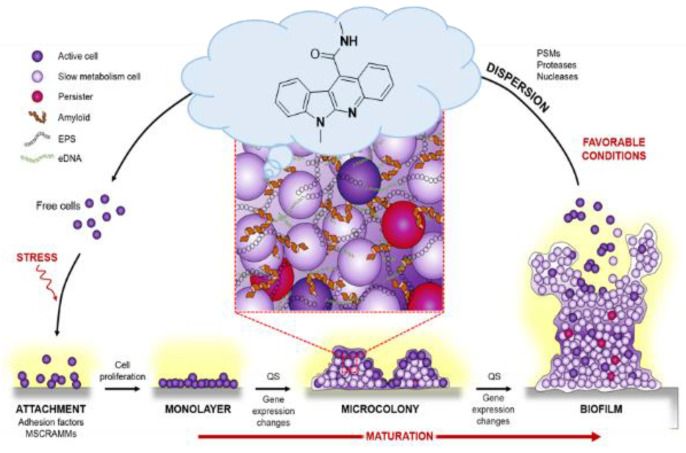
Impact of new indolo[2,3-*b*]quinolines on biofilm life cycle.

As part of our ongoing program dealing with the synthesis of complex indole-heterocycles of therapeutic interest, we have previously investigated a tandem reaction to see whether a 5-exo-trig cyclization could be innovatively combined with a radical promoted smiles rearrangement [104]. Because of interest in the indoloquinoline scaffold for drug discovery (Figure 24), we studied the preparation of new indolo[2,3-*b*]quinoline derivatives by this domino process, including radical smiles rearrangement, and we evaluated the antimicrobial characteristics of these tetracyclic derivatives as well as their capacity to prevent biofilm formation [105,106]. Promising results have been obtained in combination with ciprofloxacin.

### 4.18. Supramolecular Enhancement of Natural 14–3-3 Protein Ligands (P18)

**Xavier Guillory ^1^, Inesa Hadrovic ^2^, Pim J. de Vink ^1^, Luc Brunsveld ^1^, Thomas Schrader ^2^, Christian Ottmann ^1^**

^1^ Laboratory of Chemical Biology, Eindhoven University of Technology (TU/e), The Netherlands

^2^ Department of Chemistry, University of Duisburg-Essen, Germany; xavier.guillory@univ-rennes1.fr

**Figure 25:**
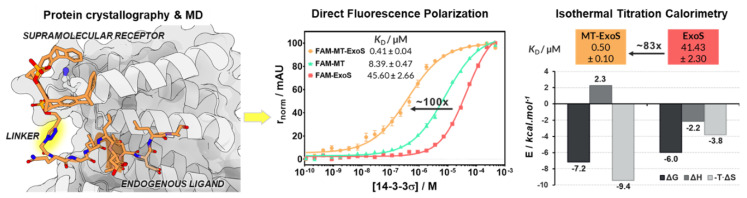
Crystal structure and biophysical characterization of the ditopic MT-ExoS ligand.

Selective recognition of proteins and modulation of their interactions represents an unprecedented opportunity for pharmacological innovation, but the rational design of protein–protein interaction (PPI) modulators remains challenging, mostly due to the different structural characteristics of protein interfaces compared to traditional drug targets [107]. Supramolecular systems can provide orthogonal molecular elements for enhancing affinity and selectivity in protein recognition and modulation by targeting protein elements such as side chains and peptide motifs [108]. Combining synthetic supramolecular elements with peptide recognition motifs would therefore provide an elegant and unique entry for the development of such improved tool compounds, and we report here a first successful proof of concept (Figure 25) [109].

Here, we show for the first time the interfacing of a synthetic supramolecular element (lysine-specific molecular tweezers) with an ExoS peptide recognition motif to furnish a powerful ditopic 14-3-3 ligand exhibiting up to a 100-fold improved affinity. X-ray crystal structure elucidation provided unique molecular insight into the binding mode and fully aligned with molecular dynamics simulations. Together, these data highlight that a short but flexible linker maintains enough degrees of freedom for favorable entropy contributions, while allowing both elements to occupy exactly the same binding site. Fluorescence polarization and isothermal titration calorimetry showed that the combination of both ligands shifts the KD into the nanomolar regime (KD ~400–500 nM), indicating an 80 to 100-fold stronger binding as compared to the native peptide.

### 4.19. Taking Back Control of the Immune System (P19)

**Dee Hayward, Prof Mark Searcey, Andrew Beekman**

School of Pharmacy, University of East Anglia, Norwich Research Park, NR4 7TJ Norwich, UK; d.hayward@uea.ac.uk

Cancer immunotherapy has emerged at the forefront of treatments against cancer. Immunotherapy provides specificity for affecting cancer cells, improving the side effects and potential for long-term remission through immunomemory [110]. Tumors use immune checkpoints to evade immunosurveillance by down-regulating molecules that stimulate T cells while up-regulating molecules which inhibit T cell activation. By blocking the interaction between the checkpoint molecule and its ligand, T cells will be activated to eliminate tumor cells. Immunotherapy provided a turning point in oncology, transforming treatment efficacy with a more targeted approach than conventional treatments. Programmed cell death protein 1 (PD-1) and programmed cell death ligand 1 (PD-L1) are well-described checkpoint proteins that have been successfully targeted with monoclonal antibodies [111,112].

Small molecules offer an inexpensive alternative to antibodies to modulate the protein–protein interaction (PPI) between PD-1 and PD-L1, although no small molecules have demonstrated proven binding to either protein yet. Bristol Myers Squibb reported two cyclic peptides (named peptide 57 and peptide 71) that offer an alternative solution showing tight binding to PD-L1 in the PD-1 binding site. There are structural benefits if cyclic peptides open avenues of information about hotspot targeting of PPIs. Through peptide-directed binding, these cyclic peptides will be used as scaffolds to create small molecule drug leads while maintaining the original binding properties [113]. The synthesis of cyclic peptides 57 and 71 will be discussed alongside the next steps towards peptide-directed binding to synthesize novel small molecule inhibitors of PD-1/PD-L1, including the tetrazine synthesis achieved so far.

### 4.20. Chitosan-Based Electrospun for Wound-Healing Applications (P20)

**Oana Maria Ionescu ^1^, Andreea Iacob ^1^, Maria Drăgan ^1^, Teodora Iurascu ^1^, Natalia Simionescu ^2^, Lenuța Profire ^1^**

^1^ Department of Pharmaceutical Chemistry, Faculty of Pharmacy, University of Medicine and Pharmacy Gr. T. Popa of Iasi, 16 University Street, Iasi 700115, Romania

^2^ Centre of Advanced Research in Bionanoconjugates and Biopolymers, Petru Poni Institute of Macromolecular Chemistry, 41A Grigore Ghica Voda Alley, 700487, Iasi, Romania; ionescu_mariuca@yahoo.com; oana-maria.dc.ionescu@d.umfiasi.ro

Nanofibers are sub-micron-scale fibers, with a diameter range between 10 and 100 nm. Their characteristic aspect ratio (more than 100) and high porosity are elements to look forward to attaining when designing the experiment and formulating the starting solution [114,115]. Chitosan nanofibers are described as artificial extracellular matrices (ECM) due to the resemblance of the polymer (CS) and the glycosaminoglicans of the natural ECM. The association of chitosan and biologically active molecules is of benefit due to chitosan’s properties as a potential drug enhancer [115,116]. Polyethylene oxide (PEO) is an amphiphilic, water-soluble compound. It is a biocompatible polymer used not only in cosmetic formulations, but also in the biomedical area.

Propolis extract (7.5% *v*/*v*), L-arginine HCl (5% *w*/*v*), Manuka honey (7.5% *w*/v), and propolis–*Calendula officinalis* extract, respectively, were dissolved in a mixture (1:1 volume ratio) of polyethylene-oxide (2% *w*/*v*) and chitosan (3% *w*/*v*) acetic acid solution (50% *v*/*v*). Electrospinning parameters were constant during the experiments, with a tip-to-collector distance of 28 cm, an infusion rate of 0.7 mL/hour, and a voltage below 18 kV. Four antioxidant tests (DPPH, ABTS, FRAP, phosphomolybdate assay) were performed to assess the best antioxidant matrix. Swelling kinetics, porosity degree and cytocompatibility are yet to be determined.

By adding the additives to the polymeric solution, the fiber diameter increases. There is an important link between viscosity and superficial tension and obtaining proper, smooth nanofibers. The chitosan-based nanofibers loaded with propolis–*Calendula* extract showed the best antioxidant potential in all four antioxidant tests. *Calendula* has also been traditionally used in dermatological issues and has proven beneficial in modern applications too. In this work, we successfully developed smooth, continuous, randomly oriented L-arginine/Manuka honey/Propolis/*Calendula officinalis* nanofibers, providing a new option for developing wound dressings.

### 4.21. Design, Synthesis, Bioactivity, and Molecular Modelling Studies of Novel Heterocyclic Compounds with Antileishmanial Activity (P21)

**Huseyin Istanbullu, Gulsah Karakaya, Gulsah Bayraktar, Hasan Akbaba, Nami Ege Perk, Bilge Debelec-Butuner, Kor Yereli, Ahmet Ozbilgin, Vildan Alptuzun**

Department of Pharmaceutical Chemistry, Faculty of Pharmacy, Izmir Katip Celebi University, 35620, Cigli, Izmir, Turkey; tel: 0090 232 329 3535 / 6111; huseyin.istanbullu@ikc.edu.tr

**Figure 26:**
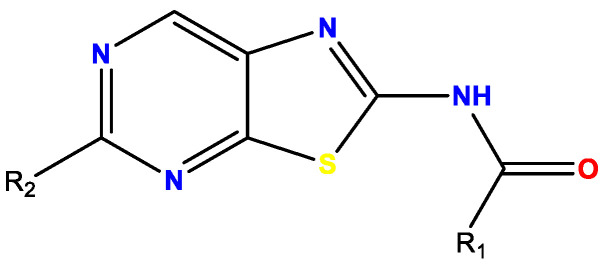
General formula of synthesized compounds.

Leishmaniasis is a major health problem in the world and classified among the neglected diseases. With the purpose of discovering novel and potent antileishmanial compounds, we designed and synthesized a series of compounds bearing a thiazolopyrimidine core (Figure 26), a classical isostere of pteridine ring which is a structural part of the native substrate of *Leishmania major* Pteridine Reductase 1 (*Lm*PTR1) enzyme [117].

Compounds’ in vitro antiparasitic activity on *L. tropica* and *L. infantum* was evaluated. Some of the compounds were selected to be tested for their in vitro DHFR inhibition activity and *Lm*PTR1 inhibitor activity [118]. Cytotoxic properties of the promising compounds were determined on RAW 264.7 murine macrophage cell line using WST-1 protocol [119]. Additionally, in vivo amastigote activity of these compounds was also investigated. Taken together, these data suggest that the introduced novel scaffold has a potential to be a lead structure in antileishmanial drug discovery.

### 4.22. Recombinant Synthesis of Human Trefoil Factor 2 Protein (P22)

**Kirtikumar B. Jadhav, Patrick Vellan, Gerhard Niederacher, Christian F.W. Becker, Markus Muttenthaler**

Institute of Biological Chemistry, University of Vienna, Währinger str. 38, 1090 Vienna, Austria; kirtikumar.jadhav@univie.ac.ato

Human trefoil factor 2 (hTFF2) belongs to an important family of peptides containing a well-structured trefoil domain [120]. hTFF2 is secreted into the gastrointestinal tract, where it plays important role in protecting and repairing the mucosa; it thus holds therapeutic promise for the treatment of chronic gastrointestinal disorders [121]. hTFF2 contains 106 amino acid residues (15Asn glycosylated) and displays two trefoil domains formed by 7 disulfide bonds. Its 3D structure, mode of action, and target receptor remain unknown, as only limited amounts of hTFF2 can be obtained from human tissue extraction.

**Figure 27:**
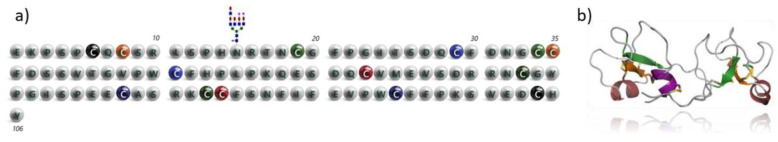
hTFF2: (**a**) sequence, (**b**) structure modelled after homologous porcine TFF2.

Here, we describe a yeast expression system designed to produce hTFF2 and its glycosylated and 15N-enriched analogues for physiological, biochemical, and spectroscopic studies (Figure 27). We designed the hTFF2 gene encoding a fusion protein, constructed recombinant plasmids, and optimized conditions for protein expression. The secreted hTFF2 was found in a glycosylated and non-glycosylated form in S. cerevisiae. We also produced a 15N-enriched analogue of hTFF2 to facilitate NMR structure determination. Furthermore, we also describe our semi-synthesis approach to synthesize hTFF2 protein by expressed protein ligation using an E. coli-based expression system. Access to large quantities and the 3D structure of hTFF2 will help to elucidate its mode of action in gastrointestinal protection and wound healing.

### 4.23. The Royal Jelly Fatty Acid 10h2da Inhibits Migratory and Invasive Potential of Colorectal Cancer Cell Lines (P23)

**Milena M. Jovanović ^1^, Dragana S. Šeklić ^2^, Jelena D. Rakobradović ^3^, Snežana D. Marković ^1^**

^1^ Department for Biology and Ecology, Faculty of Science, University of Kragujevac, Radoja Domanovića 12, Kragujevac, Serbia

^2^ Department of Natural Sciences, Institute for Information Technologies Kragujevac, University of Kragujevac, Jovana Cvijića bb, Kragujevac, Serbia

^3^ Institute for Oncology and Radiology of Serbia, Pasterova 14, Belgrade, Serbia; milena.jovanovic@pmf.kg.ac.rs

Colorectal cancer is among the most frequent cancers, whereas migration and invasion of cancer cells is particularly problematic in cancer treatment. Natural products can ameliorate standard chemotherapeutical approaches, like the unique bee product royal jelly fatty acid, trans-10-hydroxy-2-decenoic acid (10H2DA). We analyzed the anticancer effects of 10H2DA on colorectal cancer cell lines (HCT-116 and SW-480). Treatment effects on viability of tested cells was evaluated using MTT test [122], migratory potential was determined by Wound healing assay [123], invasive potential was explored by Transwell assay with modifications, and expression of promigratory proteins N-cadherin, Vimentin, and anti-invasive marker Snail was measured by immunofluorescence [122]. Cells were treated with 10H2DA in the concentration ranges 0.1, 1, 10, 50, 100, and 500 µM; as for other analysis, two selected doses (10 and 100 µM) were applied. All assays were performed 24 h after treatment. Results showed no significant cytotoxic effect of 10H2DA on both cell lines, while treatment significantly inhibited migratory/invasive potential of HCT-116 cells. The observed notable decrease of N-cadherin and Vimentin expression is in correlation with the antimigratory effect of treatment, while the lowered level of Snail confirms anti-invasive activity of 10H2DA on these cells. Royal jelly acid also induced a strong antimigratory effect on SW-480 cells with reduction of promigratory markers expression. Invasive potential of treated SW-480 cells was notably reduced, which could be elucidated by significantly lowered expression of Snail. In conclusion, our report indicates the pronounced antimigratory/invasive in vitro potential of 10H2DA on two colorectal carcinoma cell lines; thus, it should be intensively investigated in future.

### 4.24. Study of the Interactions of Caffeine-Derived Pt(II) and Pd(II) Complexes with Important Biomolecules (P34)

**Snežana Jovanović-Stević ^1^, Angelina Petrović ^2^, Jovana Bogojeski ^2^,^b^ Biljana Petrović ^2^**

^1^ University of Kragujevac, Institute for Information Technologies, J. Cvijica bb, Kragujevac, Serbia

^2^ University of Kragujevac, Faculty of Science, R. Domanovića 12, Kragujevac, Serbia; snezanaj@kg.ac.rs

Based on the report of the World Health Organization (WHO), cancer represents the second leading cause of death [124]. The application of transition-metal-based complexes as chemotherapeutics has been presented throughout history [125]. The investigation of the interactions between complexes and DNA constituents, DNA, and proteins is of crucial interest for understanding the mechanism of their action and for design of the pharmacologically effective drug. The interactions of [Pd(caffeine)_2_Cl_2_] and [Pt(caffeine)_2_Cl_2_] (caffeine = 1,3,7-trimethylxanthine) complexes with calf thymus DNA (CT-DNA) and human serum albumin (HSA) were investigated by fluorescence spectroscopy. Fluorescence-quenching measurements with DNA were performed in the presence of ethidium bromide (EB) and Hoechst dye 33258 (Hoe). The complexes have a strong ability to react with CT-DNA, suggesting intercalation and more preferable minor groove binding. High values of binding constants indicate a good binding affinity of complexes towards HSA.

### 4.25. Synthesis of 1,4-Dihydropyrazolo[4,3-B]Indoles via Intramolecular C(Sp^2^)-N Bond Formation Involving Nitrene Insertion, Dft Study, and Their Anticancer Assessment (P25)

**Manpreet Kaur, Raj Kumar**

Laboratory for Drug Design and Synthesis, Department of Pharmaceutical Sciences and Natural Products, Central University of Punjab, Ghudda, 151401 Bathinda, India; sidhukaur0013@gmail.com

**Figure 28:**
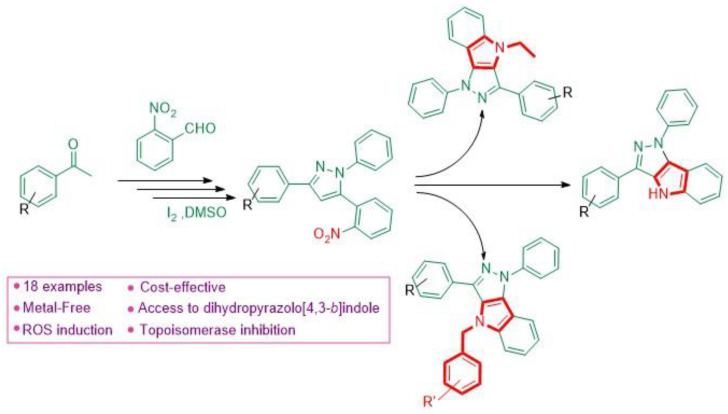
Design of target compounds.

Nowadays, C-N bond formation has been increasingly used to construct diverse N-heterocycles with various applications in pharmaceuticals, supramolecular chemistry, and so on. We herein for the first time have reported a new synthetic route for 1,4-dihydropyrazolo[4,3-*b*]indoles via deoxygenation of o-nitrophenyl-substituted N-phenyl pyrazoles, and subsequent intramolecular C(sp^2^)-N bond formation under modified microwave Cadogan condition [126]. This method exhibits a good substrate scope, and it allows access to NH-free as well as N-substituted fused indoles, which can present a potential utility in pharmaceutical as well as supramolecular chemistry applications. DFT calculations reveal the involvement of a nitrene intermediate responsible for the insertion into the C-H bond of the pyrazole ring during reductive cyclization reaction. Further, from the biological evaluation it was found that the synthesized compounds exhibited cytotoxicity at low micromolar concentration against various cancer cell lines such as A549, HCT-116, MDA-MB-231, and MCF-7; induced ROS generation; and altered the mitochondrial membrane potential of highly aggressive MDA-MB-231 cells (Figure 28).

### 4.26. Synthesis of T20K Immunosuppressive Cyclotide (P26)

**Lukas Kogler ^1^, Roland Hellinger ^2^, Kirtikumar B Jadhav ^1^**

^1^ Institute of Biological Chemistry, University of Vienna, Währinger str. 38, 1090 Vienna, Austria

^2^ Center for Physiology and Pharmacology, Medical University of Vienna, Währinger str. 13a, 1090 Austria; lukas.kogler1@gmail.com

**Figure 29:**
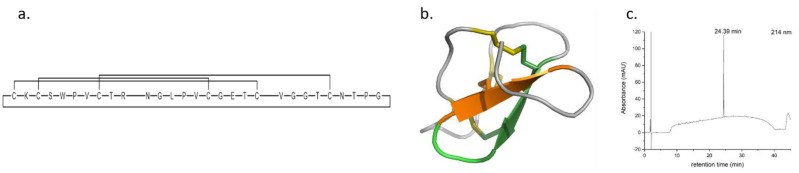
T20K (**a**) sequence and (**b**) crystal structure (PDB: 1NB1) and (**c**) HPLC chromatogram of pure T20K.

T20K is an immunosuppressive cyclotide derived from the naturally occurring plant peptide katala B1. It has been shown to suppress T-lymphocytes in an IL-2 dependent pathway. T20K is currently in phase I clinical trials for the treatment of multiple sclerosis (MS), a neurodegenerative disease driven by autoreactive T cells [127]. Besides interesting bioactivity, cyclotide T20K also features unique chemical features. It is a cyclic peptide composed of 29 amino acid residues and 3 disulfide bonds, referred to as the cyclic cysteine knot motif (Figure 29) [128]. These unique structural features confer a high chemical, enzymatic, and thermal stability. This makes them good potential candidates for drug development (e.g., molecular grafting and receptor ligand design). Here, we describe the comparison of two synthetic strategies to produce T20K in sufficient quantities.

We chose to retro-synthetically disconnect cyclic T20K between Gly11 and Gly12 for the first strategy, whereas the second strategy involved Gly18–Cys19 retrosynthetic disconnection. For the first strategy, side-chain-protected linear peptide was cyclized between Gly11 and Gly12, whereas the second strategy took advantage of native chemical ligation (NCL) to effect cyclization. Linear peptides were synthesized by Fmoc SPPS on an automated synthesizer. Upon cyclization, the peptides were folded under redox conditions to form thermodynamically stable T20K. HPLC analysis with the natural product confirmed the correct disulfide connectivity. This synthetic access to large quantities of T20K would help us elucidate its molecular mode of action in multiple sclerosis.

### 4.27. Design and Synthesis of Non-Covalent Imidazo[1,2-a]quinoxaline-Based Inhibitors of EGFR and Their Anticancer Assessment (P27)

**Manvendra Kumar, Raj Kumar**

Laboratory for Drug Design and Synthesis, Department of Pharmaceutical Sciences and Natural Products, School of Health Sciences, Central University of Punjab, Bathinda 151401, India; pharma.manvendra003@gmail.com

**Figure 30:**
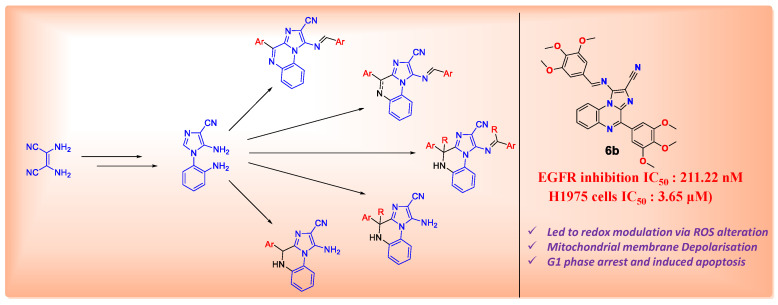
Target compounds **5–10**.

A series of non-covalent imidazo[1,2-*a*]quinoxaline-based epidermal growth factor receptor (EGFR) inhibitors were designed and synthesized [129,130]. EGFR inhibitory assessment (against wild type) data of compounds has showed compounds **6b** (IC_50_ = 211.22 nM), **7h** (IC_50_ = 222.21 nM), **7j** (IC_50_ = 193.18 nM), **9a** (IC_50_ = 223.32 nM), and **9c** (IC_50_ = 221.53 nM) as potent EGFRWT inhibitors, which were comparable to erlotinib (221.03 nM), a positive control. Furthermore, compounds exhibited outstanding anti-proliferative activity when tested against cancer cell lines A549, a non-small-cell lung cancer (NSCLC); HCT-116 (colon); MDA-MB-231 (breast); and gefitinib-resistant NSCLC cell line H1975 harboring EGFRL858R/T790M. Compound **6b** (Figure 30) showed considerable inhibitory potential against gefitinib-resistant H1975 cells (IC_50_ = 3.65 μM) as compared to gefitinib (IC50 > 20 μM). Moreover, molecular docking disclosed the binding mode of the **6b** to the domain of EGFR (wild type and mutant type), indicating the basis of inhibition. Further, its effects on redox modulation, mitochondrial membrane potential, cell cycle analysis, and cell death mode in A549 lung cancer cells were also reported.

### 4.28. Pyrazolones as Inhibitors of Immune Checkpoint Inhibitors Blocking Pd-1/Pd-L1 Interactions (P28)

**Raphaël Le Biannic ^1^, Natascha Leleu-Chavain ^1^, Romain Magnez ^2^, Frederique Klupsch ^1^, Morgane Tardy ^2^, Hassiba El Bouazzati ^2^, Gerard Vergoten ^1^, Nicolas Renault ^1^, Christian Bailly ^3^, Bruno Quesnel ^2^, Xavier Thuru ^2^, Régis Millet ^1^**

^1^ Univ. Lille, Inserm, U1286-INFINITE-Lille Inflammation Research International Center, ICPAL, 3 rue du Professeur Laguesse, 59000 Lille, France

^2^ Univ. Lille, CNRS, Inserm, CHU Lille, UMR9020–UMR1277-Canther–Cancer Heterogeneity, Plasticity and Resistance to Therapies, 1 place de Verdun, 59000 Lille, France

^3^ Oncowitan, Lille, France; raphael.le-biannic@univ-lille.fr

**Figure 31:**
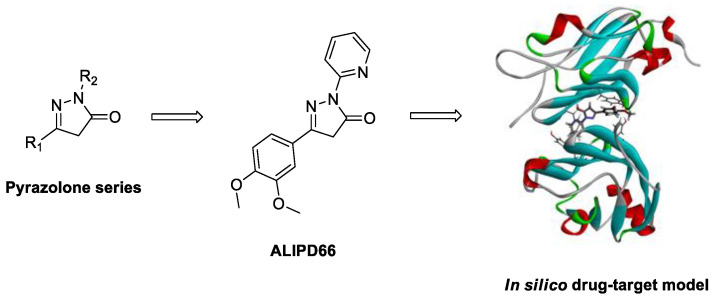
Example of one of the lead molecules of the pyrazolone series and its in silico drug-target model.

Immunotherapy has become a leading strategy to fight cancer. Over the past few years, immunotherapies using checkpoint inhibitor monoclonal antibodies (mAbs) against programmed death receptor 1 (PD-1) and programmed death ligand 1 (PD-L1) have demonstrated improved survival compared with chemotherapy [131]. We describe the identification and characterization of an innovative series of synthetic compounds (patented) endowed with nanomolar activity against PD-L1. Compounds’ properties were characterized using several biophysical techniques including microscale thermophoresis (MST) and fluorescence resonance energy transfer (FRET) measurements. In vitro, selected small molecules demonstrate a high affinity for human PD-L1, potently disrupt the PD-L1:PD-1 interaction, and inhibit Src homology region 2 domain-containing phosphatase (SHP2) recruitment to PD-1. More than 50 molecules from the pyrazolone family have been synthesized and a dozen highly potent “PD-L1 silencing compounds” have been identified, based on in vitro measurements. Structure–activity relationships have been defined and an in silico drug-target model supporting the mechanism of action has been built (Figure 31).

### 4.29. Benzo[D]Thiazol-2(3h)-Ones as New Potent Selective Cb2 Agonists with Anti-Inflammatory Properties (P29)

**Natascha Leleu-Chavain ^1^, Davy Baudelet ^1^, Valéria Moas Heloire ^1^, Diana Escalante Rochas ^1^, Madjid Djouina ^2^, Amélie Barczyk ^1^, Mathilde Body-Malapel ^2^, Pascal Carato ^3^, Régis Millet ^1^**

^1^ Univ. Lille, Inserm, ICPAL, U995-LIRIC-Lille Inflammation Research International Center, F-59000 Lille

^2^ Univ. Lille, Inserm, CHU Lille, U995-LIRIC-Lille Inflammation Research International Center, F-59000 Lille

^3^ Université de Poitiers, UFR de Médecine et de Pharmacie, IC2MP-CNRS 7285, 6 rue de la Milétrie Bât D1-TSA 5115 86073 Poitiers; natascha.leleu@univ-lille.fr

**Figure 32:**
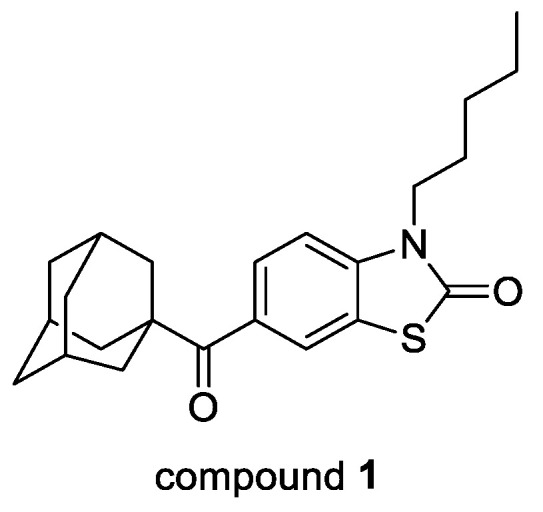
Chemical structure of compound **1**.

The high distribution of CB_2_ receptors in immune cells [132] suggests their important role in the control of inflammation [133]. CB_2_ selective agonists have the capability to modulate inflammation without triggering psychotropic effects due to the activation of CB_1_ receptors [134,135]. Therefore, there is growing evidence to consider this receptor as an attractive therapeutic target. More specifically, CB_2_ receptors’ activation represents a very promising strategy to treat gastrointestinal inflammatory diseases. In this work, we designed new selective CB_2_ agonists based on a 2-oxo-2,3-dihydro-1,3-benzothiazolinone scaffold [136]. Structure–activity relationships were studied from a series of 22 compounds. From these pharmacomodulations, we identified the importance of having both a bulky aliphatic group attached to the ketone at position 6 and an alkyl chain at *N*3 position of the heterocycle. This drug design project led to the discovery of a very potent and selective CB_2_ agonist in the nanomolar range (compound **1, Figure 32**) able to counteract colon inflammation in vivo.

### 4.30. Synthesis and Antipseudomonal Activities of New Iron Chelator–Ciprofloxacin Conjugates (P30)

**Pauline Loupias ^1^, Nicolas Taudon ^2^, Alexandra Dassonville-Klimpt ^1^, Pascal Sonnet ^2^**

^1^ AGIR, UR 4294, UFR de Pharmacie, UPJV, 1 rue des Louvels, 80037 Amiens, France

^2^ Unité de Toxicologie Analytique, IRBA, 91223 Brétigny-sur-Orge, France; pauline.loupias@u-picardie.fr

**Figure 33:**
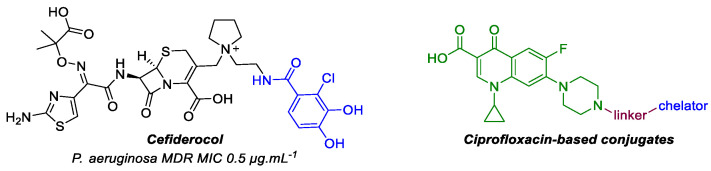
Structures of cefiderocol and ciprofloxacin-based conjugates.

Each year, antibiotic-resistant germs cause at least 700,000 deaths worldwide. Among them, the Gram-negative bacterium *P. aeruginosa* has the highest burden of healthcare-acquired infections in intensive care units and belongs to the first list of antibiotic-resistant “priority pathogens” described by the WHO [137]. In Europe, 31.8% of the cases reported to EARS-Net in 2019 were resistant to at least one antimicrobial group [138]. Alarmingly, the number of pan-drug-resistant specimens, untreatable with any of the antipseudomonal antibiotics available in the clinic, has increased [139]. The double-layered cell envelope of *P. aeruginosa* is responsible for a decreased penetration and low activity of many antibiotics. An innovative idea to bypass this barrier and restore the activity of conventional antibiotics, such as β-lactams or fluoroquinolones, is the exploitation of the siderophore-dependent active iron uptake with a “Trojan horse” strategy. In this approach, an antibiotic is chemically coupled to a natural or synthetic siderophore molecule, which forms an iron complex, thereby enhancing its active transport through bacteria outer membrane receptors (OMR). Cefiderocol, a catecholate cephalosporin conjugate using the “Trojan horse” strategy was recently approved in the USA (2019) and in Europe (2020) and is indicated for the treatment of MDR Gram-negative bacteria including *P. aeruginosa* (Figure 33) [140]. Herein, we will describe the synthesis and the antipseudomonal activities of ciprofloxacin-based conjugates bearing iron chelator moieties (catechol or hydroxypyridinone), via a linker, cleavable or not.

### 4.31. Investigation of CA-4 Metabolism and Related β-Lactam Analogues in Chemoresistant HT-29 Colon Cancer Cells (P31)

**Azizah M. Malebari ^1,2^, Mary J. Meegan ^2^**

^1^ Department of Pharmaceutical Chemistry, College of Pharmacy, King Abdulaziz University, Jeddah 21589, Saudi Arabia

^2^ School of Pharmacy and Pharmaceutical Sciences, Trinity College Dublin, Trinity Biomedical Sciences Institute, 152-160 Pearse Street, Dublin 2 DO2R590, Ireland; amelibary@kau.edu.sa

**Figure 34:**
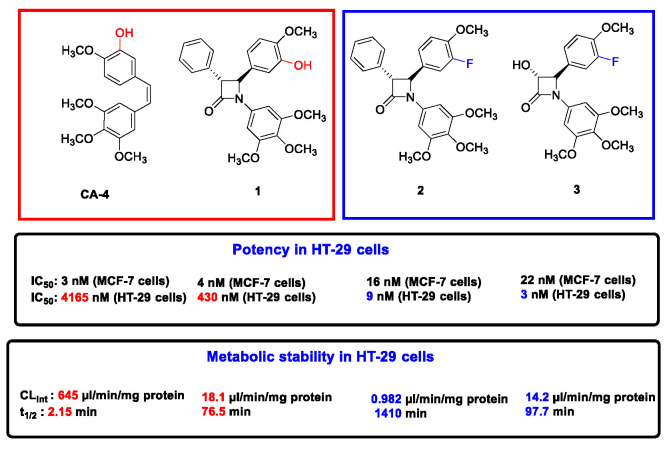
Metabolic stability in HT-29 cells of CA-4 and its representative β-lactam analogues.

Drug resistance is a common cause of the failure of chemotherapeutic agents to achieve cytotoxicity responses in human malignant disease. Drug inactivation by metabolism within tumor cells is recognized as an important mechanism of drug resistance [141]. Glucuronidation is a major route for the metabolic inactivation of many drugs and also endogenous substances. Combretastatin-A4 (CA-4) undergoes direct glucuronidation in the presence of UGTs at the meta-hydroxy group of the B-ring and could cause an inherent resistance in HT-29 colon cancer cells [60,142]. Here, we assessed the strategic deletion of the ring B hydroxyl group to produce CA-4 analogues that are equally effective in cancer cells expressing UGTs as compared to those expressing little or undetectable levels of UGTs, offering a simple solution to overcoming resistance associated with glucuronidation of CA-4 (Figure 34). These compounds play a dual role by improving the stability by blocking the isomerization of the CA-4 olefin bridge and overcoming the resistance in HT-29 colon cancer cells by improving the metabolic stability. The stability of CA-4 and its β-lactam analogue in HT-29 cells in the absence and presence of many different inhibitors of UGT enzymes (propofol, Borneol, bile acids, U0126 and 4-nitrophenol) was examined. Collectively, these data suggest a key role of UGT in mediating the resistance effect of CA-4 in HT-29 cells and provides a rationale to improve the therapeutic efficacy of CA-4 and its related analogues.

### 4.32. Using Metabolic Glycoengineering for Targeted Treatment of Cancer (P32)

**Madonna Mitry ^1,2^, Francesca Greco ^1^, Helen Osborn ^1^**

^1^ School of Pharmacy, University of Reading, Whiteknights, Reading RG6 6AD, UK

^2^ Dept of Pharmaceutical chemistry, Faculty of Pharmacy, Ain Shams University, Cairo 11566, Egypt; m.m.adeebmitry@pgr.reading.ac.uk

The main limitation of conventional chemotherapies is the poor selectivity to cancer cells, which leads to serious side effects. We aim to develop a novel selective targeting method to address this challenge. This will exploit a specific property of cancer cells, namely the overexpression of certain surface glycans called tumor-associated carbohydrate antigens, or “TACAs” [143]. The biosynthesis of these surface glycans can be intercepted through the exposure of cells to unnatural monosaccharide precursors in order to engineer unnatural functionalities on the cancer cells’ surfaces in a process called metabolic glycoengineering [144]. Hence, by using azide-modified monosaccharides, the azide functionality will be expressed on the surface of the cancer cells. This can then act as a bio-orthogonal label that can be subsequently targeted by phosphine-modified prodrugs. The bio-orthogonal Staudinger ligation reaction between the azide on the tumor cell and the phosphine in the prodrug will lead to the prodrug activation specifically at the tumor site.

As a proof of concept, we have investigated the feasibility of the Staudinger ligation reaction for prodrug activation through an HPLC-monitored release study. This involved 9-azido-*N*-acetyl neuraminic acid (as an example of an azide-modified sialic acid that will be expressed on the surface of the cancer cells after the metabolic glycoengineering) and 4-nitrophenyl 2-(diphenylphosphino) benzoate (as an example of a phosphine-modified prodrug) in an aqueous environment at 37 °C. Release of 4-nitrophenol (our model ‘drug’) reached 80% over 72 h, which confirms the potential of our proposed strategy.

### 4.33. Synthesis and Biological Evaluation of Covalent Inhibitors of Focal Adhesion Kinase against Human Malignant Glioblastoma (P33)

**Louna Mossino Diaz ^1^, Bo Li ^1^, Céline Tomkiewicz-Raulet ^2^, Xavier Coumoul ^2^, Christiane Garbay ^1^, Mélanie Etheve-Quelquejeu ^1^, Huixiong Chen ^1^**

^1^ Chemistry of RNA, nucleosides, peptides and heterocycles, CNRS UMR8601, Université Paris Descartes, 45 rue des Saints-Pères, 75006 Paris, France

^2^ Laboratoire Molécules de Communication et Adaptation des Micro-organismes, CNRS, Muséum National d’Histoire Naturelle, 63 rue Buffon 75005-Paris, France; louna.mossino@etu.u-paris.fr

Human malignant glioblastomas (GBM), which carry a poor prognosis, are highly aggressive and lethal brain cancers, with significant invasive and infiltrative features, resulting in a strong resistance to conventional treatments and high recurrence rates [145]. Current therapies such as surgical resection, radiation, and temozolomide chemotherapy remain mostly palliative because of their limited efficacy. Consequently, the survival rate of glioblastoma patients has still not improved in the last few decades. Thus, there is a necessity to find new and more efficient therapeutic solutions for malignant glioblastomas.

The focal adhesion kinase (FAK), an integrin downstream signaling mediator, is highly overexpressed in glioblastomas, thereby promoting tumor growth and invasion, and offering a promising target for novel glioblastoma therapy. Therefore, several ATP-competitive FAK inhibitors have been recently developed and their early clinical studies have shown encouraging results [146].

Here, we present the structure-based design and synthesis of a series of novel covalent inhibitors of FAK [147,148]. A cocrystal structure of the FAK kinase domain in complex with our compound revealed the inhibitor binding mode within the ATP binding site and confirmed the covalent linkage between the targeted Cys427 of the protein and the inhibitor. Biochemical characterization of our inhibitors showed a time-dependent inhibition of FAK kinase in vitro and a reversible/irreversible inhibition of the autophosphorylation of FAK. The biological evaluation of these compounds has proven their inhibitory potency against FAK enzymatic activity with IC_50_ values in the nanomolar range and anti-proliferative effects on several glioblastoma cell lines. These results exhibit the potential therapeutic benefits of covalent inhibitors of FAK for the treatment of human malignant gliomas and may offer a promising new targeted therapy for human glioblastomas.

### 4.34. Optimization of a Novel Fast-Acting Transmission-Blocking Antimalarial Agent (P34)

**Laura Mourot ^1^, Marjorie Schmitt ^1^, Elisabeth Mouray ^2^, Isabelle Florent ^2^, Sébastien Albrecht ^1^**

^1^ Laboratoire d’Innovation Moléculaire et Applications, Université de Haute-Alsace, Université de Strasbourg, CNRS, 3bis rue Alfred Werner, 68093-Mulhouse, France

^2^ Toxicologie, Pharmacologie et Signalisation Cellulaire, INSERM UMR S 1124, Université Paris Descartes, 45 rue des Saints-Pères, 75006 Paris, France; laura.mourot@uha.fr

Despite significant progress in the control of malaria with a net reduction of morbidity and mortality over the past years, it remains one of the deadliest infectious diseases in the world. New drugs with broad therapeutic potential and novel modes of action to overcome emerging drug resistances are urgently needed. Key features of the next-generation antimalarial, termed single-exposure radical cure and prophylaxis (SERCaP), have been rationalized and resulted in the recommendation of a series of target candidate profiles (TCPs). Notably, TCP1 requires rapid elimination of the initial parasite burden, at least as fast as chloroquine.

**Figure 35:**
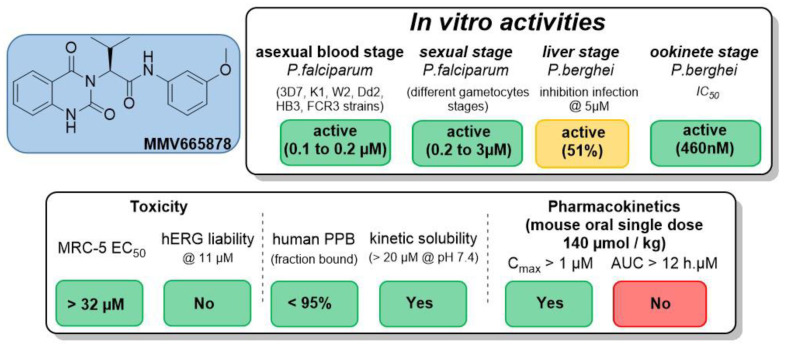
Pharmacological profile of MMV665878.

In this context, the quinazolinedione MMV665878, with its antimalarial activities against multiple life stages of Plasmodium and fast-acting and transmission-blocking activities, has great potential to deliver useful drugs for malaria parasite eradication. Moreover, this quinazolinedione-based scaffold shows a remarkable selectivity window with a low toxicity for human cells and no cardiotoxicity risk. However, pharmacokinetic issues are encountered and include moderate overall exposure and/or modest bioavailability [149], an issue probably caused by rapid metabolism and elimination. Herein, we report our progress towards the optimization of this quinazolinedione-based antimalarial series (Figure 35).

### 4.35. Ruthenium (Ii) Octahedral Complexes Featuring Pyrimidine-Like Ligands: Cytotoxicity and DNA/Protein Interaction Studies (P35)

**Alexandra-Cristina Munteanu ^1^, Mirela Mihaila ^2^, George Mihai Nitulescu ^3^, Valentina Uivarosi ^1^**

^1^ Department of General and Inorganic Chemistry, Faculty of Pharmacy, Carol Davila University of Medicine and Pharmacy, 6 Traian Vuia Str., 020956 Bucharest, Romania

^2^ Center of Immunology, Stefan S. Nicolau Institute of Virology, 285 Mihai Bravu Ave, 030304 Bucharest, Romania

^3^ Depatment of Pharmaceutical Chemistry, Faculty of Pharmacy, Carol Davila University of Medicine and Pharmacy, 6 Traian Vuia Str., 020956 Bucharest, Romania; alexandra.ticea@umfcd.ro

**Figure 36:**
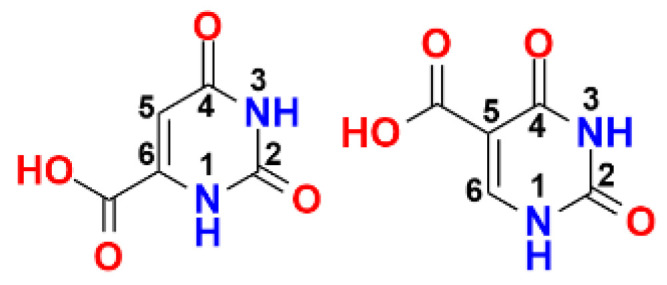
Chemical structures of orotic acid (**left**) and isoorotic acid (**right**).

Two pyrimidine-like ligands, namely orotic acid (6-carboxyuracil, H_3_Or) (Figure 1) and isoorotic acid (5-carboxyuracil, H_3_Or) (Figure 36), have been bound to rigid octahedral Ru(*N,N*)_2_ scaffolds, where (*N,N*) stands for 2,2′-bipyridine and 1,10-phenanthroline, respectively. H_3_Or is a key compound involved in the de nuovo biosynthesis of pyrimidine bases of nucleic acids in living organisms, which displays bacteriostatic and cytostatic properties, while its isomer, H_3_Or, exerts anticancer, antibacterial, and antihypertensive properties [150]. Moreover, the clinical efficacy of the Ru(II)–polypyridyl scaffold has been recently proven by TLD-1433, which has entered pivotal phase II clinical studies as a photosensitizer for intravesical photodynamic therapy against bladder cancer [151]. The physico-chemical properties and structural features of these four complexes have been investigated by spectroscopic techniques (FT-IR, UV–Vis, mass spectrometry, ^1^H and ^13^C NMR), and elemental analysis. Their cytotoxicity was tested against several cancer (breast, liver, colon, pharynx) and healthy cell lines. As a means of studying the mechanism underlying the cytotoxic effects of the complexes, interactions with calf thymus DNA were also carried out. Moreover, drug binding to serum proteins plays a very important role in drug pharmacokinetics and pharmacology, with a strong impact on absorption, distribution, metabolism, and excretion. Therefore, the binding properties of the complexes with human serum albumin and transferrin have been investigated by means of spectrophotometric studies.

### 4.36. Samarium-Doped Anatase Tio2 Nanoparticles: Synthesis, Characterization, and Synergy between Sm Rare Earth Doping and Oxygen Vacancies (P36)

**Chaima Ouled Amor ^1^, Kais Elghniji ^1^, Clarence Charnay ^2^, Elimame Elaloui ^1^**

^1^ Dept of Chemistry, Faculty of sciences of Gafsa, Gafsa, Tunisia

^2^ Institut Charles Gerhardt de Montpellier Equipe IMNO, Montpellier, France; amorchaima92@gmail.com

Semiconductor nanoparticles can absorb the entire incident light whose photon energy is greater than the band gap of semiconductor, which belongs to a broadband absorption. When rare earth ions are doped into semiconductor nanoparticles, some impurity energy levels could be formed in the host’s band structure. If there exists a certain energy transfer channel between the impurity energy levels and host’s band structure, the conduction band electrons or valence band holes that are excited by the incident light could relax to the impurity energy levels of the dopants. Then, the incident energy will be released in the form of radiative relaxation, resulting in light emission. Because rare earth ions have a wealth of energy levels of 4f-states, so the effective energy transfer between semiconductor nanoparticles and rare earth ion dopants can achieve multi-wavelength light emission. In the research field regarding RE^3+^-doped TiO_2_, the role of trivalent samarium ions (Sm^3+^) has attracted wide attention because of its good fluorescence efficiency in the visible and infrared region [152].

In order to obtain deeper information about the luminescence properties of Sm^3+^ ions in TiO_2_ nanoparticles, we systematically investigated the effects of samarium concentration on the nanoparticles’ structure and the luminescence properties of Sm^3+^, using a simple preparation method of sol–gel [153]. The materials were characterized by XRD, SEM, FTIR, TEM, and UV. The emissions of ^4^G_5/2_ → ^6^H_J_ (*J* = *5*/*2, 7*/*2, 9*/*2* and *11*/*2*) transitions of Sm^3+^ ions were observed under the excitation wavelength at 350 nm, and the emission intensity depended strongly on the doping concentration. It has been found that samarium addition into the TiO_2_ system leads to crystal expansion and matrix distortion, which indicates that some Sm^3+^ ions have entered into the matrix of TiO_2_ to replace Ti^4+^ ions. Due to substitution of Sm^3+^ ions in the Ti^4+^, oxygen vacancies are created, which generates shallow energy states which serve as electron traps in the bottom of conduction band and suppresses the recombination of excited electrons and holes.

### 4.37. Copper-Catalyzed C-H Arylation of Fused Pyrimidinones Using Diaryliodoniums Salts under Microwave Irradiation (P37)

**Alexandra Pacheco-Benichou, Corinne Fruit, Thierry Besson**

Normandie Univ, UNIROUEN, INSA Rouen, CNRS, COBRA UMR 6014, 76000 Rouen, France; alexandra.benichou@univ-rouen.fr

**Figure 37:**
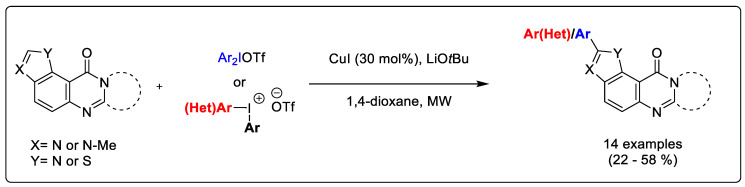
Cu-catalyzed C-H arylation process using diaryliodonium salts.

Late-stage C–H arylation of heteroarenes is a powerful tool for the synthesis of valuable scaffolds used in drug discovery. Previously, our group developed mono and bis-arylation reactions of pyrimidinones including thiazolo[5,4-*f*]quinazolin-9-(8*H*)-ones, mainly conceived as potential kinase inhibitors [154,155,156]. During the last decade, diaryliodoniums salts appeared as efficient electrophilic and non-toxic reagents for late-stage arylation [157,158]. In this context, a Cu-catalyzed C-H arylation process using diaryliodonium salts was investigated under microwave irradiation. This sustainable methodology, allowing the introduction of (het)aryl groups on the C2 atom of the thiazole moiety, was successfully extended to various fused pyrimidinones (Figure 37).

### 4.38. Sar Study of New Antikinetoplastid 3-Nitroimidazo[1,2-A]Pyridines (P38)

**Romain Paoli-Lombardo ^1^, Nicolas Primas ^1^, Sébastien Hutter ^2^, Emilie Brenot ^3^, Clotilde Boudot ^3^, Caroline Castera-Ducros ^1^, Sandra Bourgeade-Delmas ^4^, Alix Sournia-Saquet ^5^, Alexis Valentin ^4^, Nadine Azas ^2^, Bertrand Courtioux ^3^, Pierre Verhaeghe ^5^, Pascal Rathelot ^1^, Patrice Vanelle ^1^**

^1^ Aix Marseille Univ, CNRS, ICR UMR 7273, PCR, 13385 Marseille, France

^2^ Aix Marseille Univ, IHU Méditerranée Infection, UMR VITROME, 13005 Marseille, France

^3^ Université de Limoges, UMR Inserm 1094, NET, 87025 Limoges, France

^4^ Université Paul Sabatier, UMR 152 PharmaDev, 31062 Toulouse, France

^5^ Université Paul Sabatier, CNRS UPR 8241, LCC, 31077 Toulouse, France; romain.paoli-lombardo@etu.univ-amu.fr

**Figure 38:**
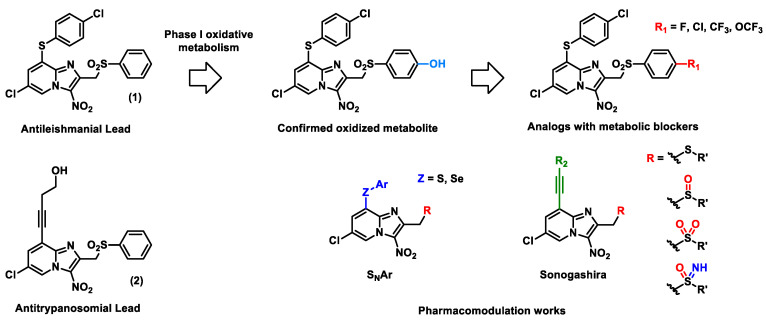
Lead compounds and new designed antikinetoplastid 3-nitroimidazo[1,2-*a*]pyridines.

Kinetoplastids are flagellated protozoans responsible for parasitic diseases in humans, including *Leishmania* spp. (leishmaniases), *Trypanosoma brucei* (sleeping sickness), and *Trypanosoma cruzi* (Chagas disease). Nearly 20 million people are infected by one of these neglected tropical diseases (NTDs) per year, causing up to 50,000 deaths. Moreover, currently available treatments have major limitations. In this context, our laboratory previously described two 3-nitroimidazo[1,2-*a*]pyridine lead compounds selectively activated by the parasitic type 1 NTR: one active in vitro against Leishmania [159], and the other against Trypanosoma [160]. However, these compounds have low solubility and poor mouse microsomal stability. Among the probable metabolites, oxidized metabolites were synthetized and confirmed to be the result of the metabolism of the lead compounds. In order to improve microsomal stability, analogues with metabolic blockers were obtained. Finally, pharmacomodulation works at the C2 position were carried out, and new molecules of therapeutic interest were synthesized [161]. The synthesis pathway and biological results of these new compounds will be presented in the communication (Figure 38).

### 4.39. New Nitric-Oxide-Releasing Indomethacin Derivatives: Synthesis and Biological Evaluation (P39)

**Lenuţa Profire ^1^, Alexandru Sava ^1^, Frédéric Buron ^2^, Sylvain Routier ^2^**

^1^ Department of Pharmaceutical Chemistry, Faculty of Pharmacy, “Grigore T. Popa” University of Medicine and Pharmacy of Iași, 16 University Street, Iasi, Romania

^2^ Institut de Chimie Organique et Analytique ICOA, CNRS UMR 7311, Université d’Orléans, Orléans, France; lenuta.profire@umfiasi.ro

Nitric-oxide-releasing non-steroidal anti-inflammatory drugs (NO-NSAIDs) are a new class of anti-inflammatory drugs consisting of a traditional NSAID to which an NO-releasing moiety has been covalently attached by a spacer [162,163]. NO is an endogenous short-lived free radical, produced in mammalian cells through nitric-oxide-synthase-mediated conversion of L-arginine to L-citruline [164]. It is known that NO has a key role in a wide variety of physiological and pathophysiological processes, such as inflammation, vasodilatation, platelet adhesion, thrombosis neurotransmission, neuronal communication, and wound healing [165,166]. In this study, we present the design and synthesis of new nitric-oxide-releasing indomethacin derivatives with 1,3-thiazolidine-4-one scaffold (NO-IND-TZDs), as a new safer and efficient multi-target strategy for inflammatory diseases. The chemical structure of synthesized derivatives was proven by NMR and mass spectroscopic analysis. The synthesized compounds were evaluated in terms of anti-inflammatory and antioxidant effects, using in vitro assays, as well as the nitric oxide (NO) release. The tested compounds showed improved radical-scavenging effects, the highest radical-scavenging effect being noted for **6n**, which contains a 2-(4-nitro-phenoxy)ethyl nitrate moiety. For this compound, the highest NO release capacity was also noted, which means it could have reduced side effects on the GI level, such as irritation, bleeding, or ulceration. Moreover, the best predicted anti-inflammatory effect, measured as BSA denaturation, was showed by **6i,** which contains a (2,6-dichloro-phenoxy)ethylnitrate moiety, which supports the good influence of chloro substituent for anti-inflammatory effects. The results of our study strongly support the potential effect of NO-IND-TZDs as a multi-target strategy, targeting the inflammation, oxidative stress, and NO release.

### 4.40. Fishing Potent Epac2 Inhibitors: An Interdisciplinary Approach (P40)

**Urooj Qureshi ^1^, Israr Khan ^2^, Sajda Ashraf ^1^, Abdul Hameed ^2^, M. Hafizur Rahman ^2^, Zaheer Ul-Haq ^1,2^**

^1^ H.E.J. Research Institute of Chemistry, International Center of Chemical and Biological Sciences, University of Karachi, Karachi-75210, Pakistan

^2^ Dr. Panjwani Center for Molecular Medicine and Drug Research, International Center of Chemical and Biological Sciences, University of Karachi, Karachi-75210, Pakistan; uroojquareshi@ymail.com; zaheer.qasmi@iccs.edu

Exchange protein activated by cAMP 2 (EPAC2) is a direct target of 3’−5’-cyclic adenosine monophosphate (cAMP), which is involved in cAMP-mediated signal transduction through activation of the Ras-like small GTPase Rap. EPAC signals play crucial roles in almost every disease related to dysfunctional signaling pathways. Therefore, EPAC represents an excellent drug target against various diseases as a signal transduction component. To date, very few EPAC inhibitors are available; however, there is still a lack of isoform-selective inhibitors due to off-target effects. Selective inhibition of one isoform leads to work on the allosteric site in EPAC2 that is distinct from the active site shared by EPAC variants [167,168]. Here, we present a site-directed approach, specifically targeting the EPAC2 isoform.

To explore the allosteric site, we applied multiple pharmacophore modeling, molecular docking, and molecular dynamics simulation techniques. Primarily, the commercially available databases NCI and Maybridge and an in-house database were screened against validated pharmacophore models. Molecules obtained were scrutinized by molecular docking to predict their binding affinities. In step-wise screening, four compounds were short-listed for stability dynamics to get deep insight into their binding mechanism. Simultaneously, inhibitory activities of these compounds were investigated to validate their effect on mice pancreatic islets. The results highlighted the efficiency of pharmacophores to access diverse chemical classes that can contribute to designing selective probes for EPAC2-associated pathologies.

### 4.41. New Mononuclear Gold(Iii) Complexes—Study of the DNA/HSA/BSA-Binding Properties (P41)

**Snežana Radisavljević ^1^, Milica Međedović ^1^, Ana Rilak ^2^, Dušan Ćoćić ^1^, Biljana Petrović ^1^**

^1^ University of Kragujevac, Faculty of Science, R. Domanovića 12, Kragujevac

^2^ University of Kragujevac, Institute for Information Technologies, J. Cvijica bb, Kragujevac; snezana.radisavljevic@pmf.kg.ac.rs

Considering that cancer is one of the major human problems, the discovery of metal-based drugs which can be used as anticancer agents represents the most important field of investigation [169]. It is already known that platinum(II) complexes have shown great impact on the treatment of different cancers, but scientists continue to make efforts to develop new drugs. The most promising new metal-based drugs are gold(III) complexes, due to their similarities with platinum(II) complexes. The main problem for usage of gold(III) complexes is their reduction to gold(I) or gold(0), but this can usually be solved with the right choice of ligands [170]. DNA is the main target for many metal-based antitumor drugs, and interactions with DNA have a major role in the development of new drugs. In addition, serum proteins are important for the regulation of osmotic pressure as well as blood pH. Consequently, interaction between human serum albumin (HSA)/bovine serum albumin (BSA) and drugs have a big impact on the development of potential drugs.

**Figure 39:**
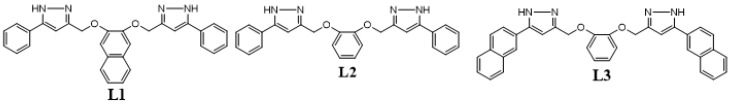
Structures of ligands.

New gold(III) complexes with the general formula [Au(N-N)Cl_2_]^+^ for complexes **1–3** (where N-N is **L1**, **L2** or **L3**), and [Au(N-N)_2_]^3+^ for complexes **4–5** (where N-N is **L1** or **L2**) were synthesized (Figure 39). The interactions of these complexes and CT-DNA were evaluated by different methods: UV–Vis spectroscopy, fluorescence spectrometry, and viscosity measurements, while interactions with HSA/BSA were investigated by fluorescence spectroscopy. The binding of all complexes with CT-DNA was confirmed with the high values of intrinsic binding constant (Kb), while further examinations confirmed that the mode of binding is groove binding. The performed experiments have shown a good ability of complexes for binding to serum albumins, especially BSA, with the value of binding constants in the optimal range.

### 4.42. Aralkylpyrimidinetriones as Growth Inhibitors of Clostridioides Difficile (P42)

**Bassant Rateb, Tony Worthington, William Fraser**

College of Health and Life Sciences, Aston University, Aston Triangle, B4 7ET Birmingham, UK; Bassant Rateb

There is a pressing clinical need for effective new antimicrobial agents against *Clostridioides difficile*, one of the UK’s leading nosocomial healthcare-associated infection pathogens. Strains of *C. difficile* have become less sensitive to the current frontline therapies metronidazole and vancomycin, with growing resistance to many other antibiotics [171]. We have prepared aralkylpyrimidinetriones (APTs), some of which are active against *C. difficile* but inactive against *Escherichia coli* and *Staphylococcus aureus.* Selective targeting of *C. difficile* would provide a significant advantage over current antibiotic treatments that also destroy various gut-colonizing bacteria and exacerbate life-threatening *C. difficile*-associated diarrhea.

Dihydroorotate dehydrogenase (DHODase) catalyzes the reversible and rate-determining fourth step of de novo pyrimidine biosynthesis. Arylidene barbiturates are known to inhibit purified DHODase from the organism *Clostridioides oroticum,* with the rate of inhibition being strongly dependent upon the electronic properties of the aryl substitutents [172,173]. Indiscriminate reactivity towards nucleophiles, poor aqueous solubility, and the risk of unwanted anxiolytic, hypnotic, and sedative properties associated with uncharged CNS-active barbiturate drugs render arylidene barbiturates poor candidates as potential anti-infective agents. We considered APTs to be surrogate substrates with potential as novel pro-drug inhibitors of DHODase that exploit the enzyme’s ability to create C=C bonds [174]. Once bound at the DHODase active site, the benzylic C-C bond in the APT prodrug may conceivably be oxidized to give an exocyclic C=C bond, generating the reactive arylidene at the DHODase active site, to which a proximal nucleophile can attach followed by deactivating protonation, leading to irreversible inhibition of the enzyme. Better aqueous solubility and closer resemblance to the enzyme’s natural substrate is predicted for the APT derivatives were ionization to generally occur at C5 (pK_a_ = 3.9) [175] under physiological conditions.

We demonstrated success in the use of uncatalyzed Knoevenagel condensation between barbituric acid and various 2-aryl-substituted acrylaldehydes in ethanol (1 h, reflux) to give moderate to good yields of diene derivatives of barbituric acid, from which APT analogues were readily prepared by reduction using sodium borohydride or palladium on charcoal. We achieved regiospecific reduction of the exocyclic double bond using sodium borohydride, whereas reduction of both double bonds to give a saturated propyl linkage resulted from use of palladium on charcoal (1 h, sonication, 2–3 atm). Representative compounds in the arylidene and APT series were evaluated and shown to have *C. difficile* growth inhibitory properties, but with reduced or no activity against *E. coli* and *S. aureus*.

### 4.43. 1,3-. Dipolar Cycloaddition of Diazo Compounds to Activated Enynes (P43)

**Sergey A. Sokov ^1,2^, Simon S. Zlotskii ^1^, Ivan S. Odin ^2^, Alexander A. Golovanov ^2^**

^1^ Ufa State Petroleum Technological University, Ufa, Russia

^2^ Togliatti State University, Togliatti, Russia; s.a.sokov.tltsu@gmail.com

**Figure 40:**
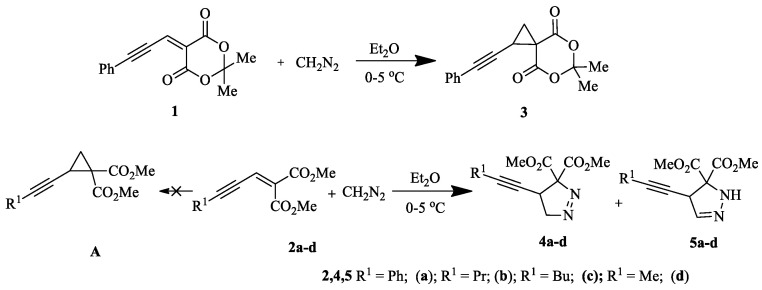
Reaction of enynes with diazomethane solution.

The reactions of 1,3-dipolar cycloaddition of diazo compounds to unsaturated compounds, such as enynones, is of great interest. As shown earlier, such interaction in the presence of catalysts can lead to the formation of furan derivatives [176]. The interaction of activated enyne structures is poorly studied. We have studied the 1,3-dipolar cycloaddition of diazomethane to the enyne derivatives of Meldrum acid **1** and dimethylmalonate **2** (Figure 40).

It was shown that compounds **1** and **2**, due to the polarization effect of CO groups, easily react with diazo compounds, specifically at double C=C bonds. In the case of the Meldrum acid derivative **1**, the reaction with an ether solution of diazomethane gives the cyclopropane derivative **3** with a yield of 80%. The reaction of compounds **2** with an ether solution of diazomethane proceeds with the formation of pyrazolines **4** and **5** in high yields (>95%). Structures **5** are minor isomers, the content of which does not exceed 10%. It should be noted that for products **4** and **5**, it is not possible to register high-resolution mass spectra, since under electrospray conditions, they decompose with the evolution of nitrogen; in this case, a peak corresponding to the molecular ion of cyclopropane is recorded. Nevertheless, the composition of these products is reliably confirmed by quantitative elemental microanalysis for carbon and hydrogen. The structure was confirmed by NMR spectroscopy.

Thus, we have shown that electron-deficient 1,3-enynes containing in the first position one or two electron-withdrawing groups react with diazomethane on a double carbon–carbon bond in the absence of a catalyst. The data obtained can further serve as the basis for the development of methods for the synthesis of polyfunctional compounds containing structural elements of nitrogen-containing heterocycles and cyclopropane.

### 4.44. Torquoselective Nazarov Cyclization Mediated by a Chiral Sulfoxide (P44)

**Xavier J. Salom-Roig, Erwann Grenet, Jean Martinez**

Institut des Biomolécules Max Mousseron (IBMM), UMR 5247, Université de Montpellier, CNRS, ENSCM, Place Eugène Bataillon, 34095 Montpellier, France; xavier.salom-roig@umontpellier.fr

**Figure 41:**
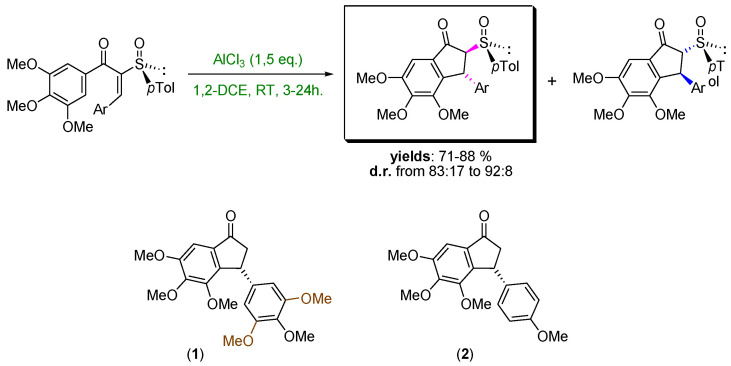
Torquoselective Nazarov cyclization and anticancer agents **1** and **2**.

We report here a Nazarov cyclization reaction of activated dienones that contain an aromatic moiety as an electron-donating group and a chiral sulfoxide that act as both an electron-withdrawing group and a chiral auxiliary. The sulfinyl group directed the torquoselectivity and AlCl_3_ was used as a promoter. Only the *trans* stereoisomers were observed. Substrates that contained activated aromatic moieties, including phenyl and aromatic heterocycles, led to the desired cyclopentenones. The potential use of this method was highlighted in the first enantioselective synthesis of the two anticancer agents **1** and **2** (Figure 41) [177,178].

### 4.45. Design and Development of Novel Urea, Sulfonyltriurea, and Sulfonamide Derivatives as Potential Inhibitors of Sphingosine Kinase 1 (P45)

**Sonam Roy ^1^, Amarjyoti Das Mahapatra ^2^, Taj Mohammad ^1^, Preeti Gupta ^1^, Mohamed F. Alajmi ^3^, Afzal Hussain ^3^, Md. Tabish Rehman ^3^, Bhaskar Datta ^2^, Md. Imtaiyaz Hassan ^1^**

^1^ Centre for Interdisciplinary Research in Basic Sciences, Jamia Millia Islamia, Jamia Nagar, 110025 New Delhi, India

^2^ Department of Chemistry, Indian Institute of Technology, Palaj, Gandhinagar, 382355 Gujarat, India

^3^ Department of Pharmacognosy, College of Pharmacy, King Saud University, 11451 Riyadh, Saudi Arabia; roysonam12@gmail.com

**Figure 42:**
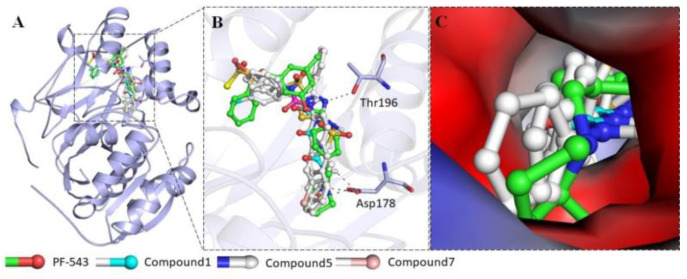
Structural representation of the binding pattern of Compounds **1**, **5**, and **7** along with PF-543 in the substrate-binding site cavity of SphK1. (**A**) Cartoon representation of SphK1 showing ligands bound to the active site cavity. (**B**) Interaction of Compounds **1**, **5**, and **7** with the SphK1 residues. (**C**) Surface model highlighting the active site pocket of SphK1.

SphK1 promotes fundamental cellular processes including cell survival, proliferation, migration, and immune function. SphK1 inhibition is considered as an attractive strategy for cancer therapeutics [179,180]. Recently discovered small-molecule inhibitors of SphK1 have been recommended in cancer therapeutics; however, the selectivity and potency of first-generation inhibitors are a great challenge [181,182]. In search of effective SphK1 inhibitors, a set of 11 novel small molecules were designed and synthesized bearing urea, sulfonylurea, sulfonamide, and sulfonyltriurea groups and screened for their inhibitory activity against SphK1. Fluorescence binding studies, isothermal titration calorimetry (ITC), enzyme inhibition assay, and molecular docking were performed to gain insights into the binding and inhibition mechanism (Figure 42).

Compounds **1**, **5**, and **7** bound to the SphK1 with a higher affinity in the sub-micromolar range and significantly inhibited its activity with IC_50_ values in the micromolar range. Molecular docking studies revealed that these compounds fit well into the sphingosine-binding pocket of SphK1 and formed a significant number of hydrogen bonds and van der Waals interactions. While urea and sulfonylurea derivatives have been reported as SphK1 inhibitors, this is the first report of a sulfonyltriurea providing the scope of SphK1 inhibition. Henceforth, compounds **1**, **5**, and **7** might be exploited as novel scaffolds for the generation of potent and selective SphK1 inhibitors that could be implicated in cancer therapeutics after the required in vivo validation.

### 4.46. Development of Chromone Carboxamides as Quorum-Sensing Inhibitors for the Treatment of Cf-Related Multi-Species Biofilms (P46)

**Jeanne Trognon ^1^, Maya Rima ^1^, Gonzalo Vera ^1^, Jean-Luc Stigliani ^2^, Barbora Lajoie ^1^, Salomé El Hage ^1^, Christine Roques ^1^, Fatima El Garah ^1^**

^1^ Laboratoire de Génie Chimique, Université de Toulouse, CNRS, INPT, UPS, Toulouse, France

^2^ Laboratoire de Chimie de Coordination, CNRS, INPT, UPS, Toulouse, France; jeanne.trognon@univ-tlse3.fr

Cystic fibrosis (CF) patients often suffer from chronic pulmonary infections caused by bacterial strains, such as Pseudomonas aeruginosa, Staphylococcus aureus, and Burkholderia cepacia, known to grow in patients’ lungs as biofilms, a more virulent and antibiotic-resistant bacterial lifestyle. A major mechanism behind biofilm formation is quorum sensing (QS), a communication system where bacterial cells produce, detect, and respond to auto-inducers, such as acyl homoserine lactones (AHLs) and alkylquinolones (AQs) in Gram-negative bacteria [183,184]. QS inhibition has been proven to be a promising strategy to control biofilms. Our group has identified a AHL analog capable of inhibiting the formation of P. aeruginosa biofilm in vitro [185,186] and reduce its virulence in vivo [187]. QS inhibition within multi-species biofilms has been less studied so far.

In view of this, we investigated the ability of the chromone scaffold to serve for the design of structural analogs and potential inhibitors of the PQS auto-inducer. We used molecular docking to predict their binding affinity with the active site of the PqsR receptor protein. Chromone carboxamides with the best binding affinity for PqsR were synthesized via straightforward routes and in goods yields. Their ability to inhibit the formation of biofilms was first evaluated on P. aeruginosa PAO1. Several compounds showed a promising anti-biofilm activity, with a significant decrease of the total adherent biomass. In parallel, we also developed a 3-species biofilm inhibition assay for the evaluation of active compounds selected on PAO1. The design, synthesis, biological evaluation on PAO1, and setup of the three-species anti-biofilm assay will be presented. Overall, results showed chromones carboxamides are a promising series for the selection of a lead compound for further development in view of improving the prevention and treatment of CF-related multi-species infections.

### 4.47. Removing Cancer’s Immortality: Targeting Telomerase (P47)

**Suzanne van Wier, Mark Searcey, Andrew Beekman**

School of pharmacy, University of East Anglia, Norwich research park, NR4 7TJ, Norwich, Norfolk, UK; s.van-wier@uea.ac.uk

One of the hallmarks of cancers is their ability to replicate limitlessly, making them immortal. In 80–90% of cancer cells this is due to the reactivation of telomerase, a protein complex which elongates telomeres at the end of chromosomes, protecting the chromosomes from degradation and preventing cell senescence.

The Cryo-EM structure of telomerase published in 2018 provided an opportunity to identify new ways to target telomerase [188]. In patients with dyskeratosis congenita, a disease characterized by shortened telomeres, the structure showed that genetic mutations are transcribed to the dyskerin–dyskerin protein–protein interaction (PPI) in telomerase. In this project, we aim to target this PPI, inhibiting the telomerase activity of cancer cells and thus removing cancer’s immortality.

We will describe the design and synthesis of a peptide derived from the dyskerin sequence at this PPI and an alanine scan of this peptide to identify the amino acids most important for binding. These peptides will be assessed for their binding affinity towards dyskerin, their α-helicity and their effect on telomerase activity.

When compared to small-molecule drugs, peptide therapeutics can have drawbacks, such as their limited stability and cell permeability. Therefore, peptide-directed binding will be used to go from peptide to small molecule inhibitor by computationally identifying fragments able to replace parts of the peptide. This method has previously been applied successfully to quickly identify hit compounds for PPIs whilst minimizing the organic synthesis and biological screening needed [189].

### 4.48. An Artemisinin-Derivative–(Nhc) Gold(I) Hybrid with Enhanced Cytotoxicity through Inhibition of Nrf2 Transcriptional Activity (P48)

**Xing Wang ^1^, Catherine Hemmert ^1^, Olivier Cuvillier ^2^, Heinz Gornitzka ^1^**

^1^ LCC-CNRS, Université de Toulouse, CNRS, UPS, Toulouse, France

^2^ Institut de Pharmacologie et de Biologie Structurale, Université de Toulouse, CNRS, UPS, Toulouse, France; xing.wang@lcc-toulouse.fr

**Figure 43:**
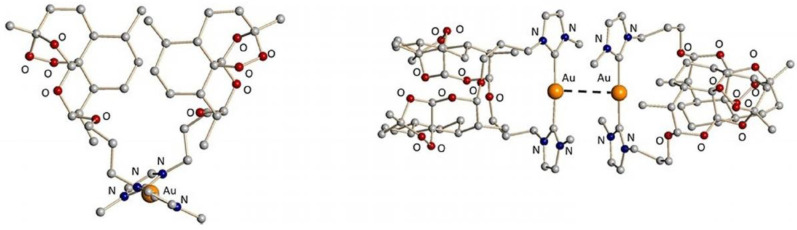
Structure of the cationic part of the complexe **2a** in the solid state. One bisNHC–gold unit looking along the C-Au-C axis on the left and dimeric arrangement on the right.

A family of hybrid complexes combining two biologically active motifs, an artemisinin derivative and a cationic bis(NHC)-gold(I) unit, has been synthesized. One of these complexes, **2a**, has been analyzed by single-crystal X-ray diffraction (Figure 43). **2a** shows strong anticancer activities on a large panel of human cancer cell models (prostate, breast, lung, liver, bladder, bone, acute and chronic myeloid leukemias) with GI50 values in the nm range, together with a high selectivity. An original and distinctive mechanism of action, that is, through inhibition of the redox antioxidant NRF2 transcription factor (strongly associated with aggressiveness and resistance to cancer therapies), has been evidenced. **2a** could remarkably sensitize to sorafenib in HepG2 liver cells, in which dysregulated NRF2 signaling is linked to primary and acquired drug resistance. **2a** also inhibited NF-κB and HIF transcriptional activities, which are also associated with progression and resistance in cancer. Our findings provide evidence that hybrid (NHC)gold(I) compounds represent a new class of organometallic hybrid molecules that may yield new therapeutic agents [190].

### 4.49. New Approaches to the Synthesis of Pyoverdine D (P49)

**Tianzhu Zhang ^1,2^, Albert Bolhuis ^1,2^, Ian M. Eggleston ^1,2^**

^1^ Department of Pharmacy & Pharmacology, University of Bath, Bath, BA2 7AY, UK

^2^ Centre for Therapeutic Innovation, University of Bath, Bath BA2 7AY, UK; tz496@bath.ac.uk

**Figure 44:**
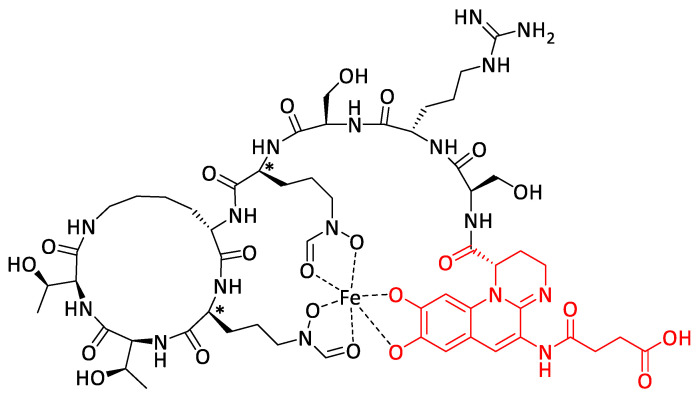
Structure of pyoverdine D. The chromophore unit is shown in red. The formylhydroxyornithine residues are highlighted by an asterisk (*).

Pseudomonas aeruginosa is a clinically opportunistic pathogen and a major threat to patients with compromised immune systems and cystic fibrosis. Iron is an essential nutrient for virtually all microbes, including P. aeruginosa, which acquires iron by the secretion of certain iron-chelating siderophores [191]. The most important of these siderophores is the cyclic peptide derivative pyoverdine D. As an endogenous siderophore, pyoverdine D is of considerable interest as it could provide a starting point for new ways to deliver toxic materials to P. aeruginosa, for instance by linking with antibiotics [192]. This requires an efficient and flexible synthetic route to be developed for this natural product. We will present our studies towards an improved modular synthesis of pyoverdine D, with a focus on the preparation of three important components—the novel amino acid, formylhydroxyornithine, which occurs twice in the pyoverdine D structure; the solid phase synthesis of the cyclic peptide unit; and the synthesis of the tricyclic “chromophore” unit (Figure 44).

### 4.50. Commercialization of Small Fluorinated Hydrophobic Groups for Lipophilicity Tuning in Drug Development (P50)

**Mariana Manso ^1^, Bruno Linclau ^1^, Steve Brough ^2^**

^1^ Chemistry, University of Southampton, Highfield, Southampton, SO17 1BJ, UK

^2^ Key Organics, Camelford, Cornwall PL32 9RA, UK; m.g.manso@soton.ac.uk

**Figure 45:**
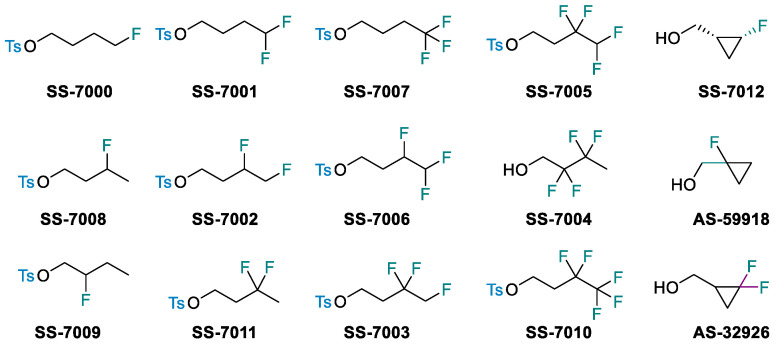
Compounds available.

Next to bioactivity, optimization of lipophilicity is crucial in drug development. Here, we describe the changes in lipophilicity using a selection of different internal and terminal fluorination patterns, motif rearrangements, and vicinal and skipped patterns on a linear alkanol and on a cyclopropylmethyl scaffold (Figure 45) [193,194]. The comparison with the corresponding non-fluorinated parents 1-butanol and cyclopropylmethanol is also shown. These trends can be replicated when these motifs are introduced in a pharmaceutical drug candidate as part of an aromatic butoxy chain [195]. The coupling reactions between these small fluorinated hydrophobic groups and the aromatic groups is typically achieved with a tosylate or triflate functional group. Hence, we offer a stock of different fluorinated 1-butanols and cyclopropylmethanols, some already activated as tosylate (which has the added benefit of reducing compound volatility).

## 5. Conclusions

The meeting attracted over 150 delegates, with remarkable growth of both the network membership and the geographical range of the attendees’ home countries, which included France, Germany, the United Kingdom, Turkey, Spain, Portugal, Ireland, Italy and Romania, but also countries outside Europe. The program comprised a discipline-leading line-up of presenters from various universities, spanning a broad range of pharmaceutical-chemistry-related topics.

Louise Cooney (YRC02, University College of Cork, Ireland) received the award for the best presentation by an early-career researcher, sponsored by *RSC Medicinal Chemistry* journal. The runner-up prize went to Eavan McLoughlin (YRC05, Trinity College, University of Dublin, Ireland), sponsored by *Pharmaceuticals*, a journal published by MDPI.

The prize for the best poster presentation by a post-doctoral researcher was awarded to Dr. Pauline Loupias (P30, University of Amiens, France), sponsored by Teledyne ISCO, and the runner-up prize was given to Dr. Xavier Guillory (P18, University of Rennes 1, France and Eindhoven University of Technology, The Netherlands), sponsored by CEM.

Sébastien Depienne (P14, University of Nantes, France) received the prize for the best poster presentation by a PhD researcher from our sponsor Key Organics, and the two runner-up prizes, sponsored by Collaborative Drug Discovery, went to Louna Mossino Diaz (P33, University Paris-Descartes, France) and Jeanne Trognon (P46, University of Toulouse, France).

Congratulations to all the awardees!

The 30th Annual GP_2_A Medicinal Chemistry Conference is scheduled to take place in person at Trinity College, Dublin, 24–26 August 2022. Our group will be delighted to welcome current and new members during this future event.

Both the GP2A and the organizing committees thank all the sponsors, namely *RSC Medicinal Chemistry*, *Pharmaceuticals* MDPI, Teledyne ISCO, CEM, Collaborative Drug Discovery, Key Organics, and Asynt, for supporting our 2021 annual conference.

## Data Availability

Data is contained in the article.
